# Orientation of mouse *H19* ICR affects imprinted *H19* gene expression through promoter methylation-dependent and -independent mechanisms

**DOI:** 10.1038/s42003-021-02939-9

**Published:** 2021-12-17

**Authors:** Hitomi Matsuzaki, Yu Miyajima, Akiyoshi Fukamizu, Keiji Tanimoto

**Affiliations:** 1 Faculty of Life and Environmental Sciences, University of Tsukuba, Tsukuba, Ibaraki Japan; 2 Life Science Center for Survival Dynamics, Tsukuba Advanced Research Alliance (TARA), University of Tsukuba, Tsukuba, Ibaraki Japan; 3 Graduate school of Life and Environmental Sciences, University of Tsukuba, Tsukuba, Ibaraki Japan

**Keywords:** Imprinting, DNA methylation, Gene expression

## Abstract

The mouse *Igf2/H19* locus is regulated by genomic imprinting, in which the paternally methylated *H19* imprinting control region (ICR) plays a critical role in mono-allelic expression of the genes in the locus. Although the maternal allele-specific insulator activity of the *H19* ICR in regulating imprinted *Igf2* expression has been well established, the detailed mechanism by which the *H19* ICR controls mono-allelic *H19* gene expression has not been fully elucidated. In this study, we evaluated the effect of *H19* ICR orientation on imprinting regulation in mutant mice in which the *H19* ICR sequence was inverted at the endogenous locus. When the inverted-ICR allele was paternally inherited, the methylation level of the *H19* promoter was decreased and the *H19* gene was derepressed, suggesting that methylation of the *H19* promoter is essential for complete repression of *H19* gene expression. Unexpectedly, when the inverted allele was maternally inherited, the expression level of the *H19* gene was lower than that of the WT allele, even though the *H19* promoter remained fully hypomethylated. These observations suggested that the polarity of the *H19* ICR is involved in controlling imprinted *H19* gene expression on each parental allele, dependent or independent on DNA methylation of the *H19* promoter.

## Abstract (web summary)

Hitomi Matsuzaki et al. test the effect of reversing the *H19*/*Igf2* imprinting control region (ICR) in mice. Their results suggest that the polarity of the ICR is involved in controlling imprinted *H19* expression in both parental alleles, dependent or independent of DNA methylation.

## Introduction

Genomic imprinting in mammals is an epigenetic phenomenon in which a subset of genes is expressed only when inherited from either the father or the mother. Because imprinted genes have diverse functions in development, growth, and behavior, disruption of their mono-allelic expression causes human diseases such as Beckwith–Wiedemann and Silver–Russell syndromes^1,2^. Imprinted genes form clusters on the genome, and their accompanying imprinted control regions (ICRs) regulate the expression patterns of multiple genes within each locus. Because most ICRs acquire DNA methylation in either the sperm or the egg, they are also called germline differentially methylated regions (gDMRs). After fertilization, differential methylation of ICRs between alleles is maintained throughout development, and, at some loci, induces allele-specific DNA methylation of secondary DMRs or somatic DMRs (sDMRs)^3,4^. Methylation of gDMRs and sDMRs regulates allele-preferential binding of transcription factors, resulting in unilateral allelic expression of genes within the locus.

Mono-allelic expression of the *Igf2/H19* gene locus is controlled by paternal allele-specific DNA methylation of the *H19* ICR, located 2–4 kb upstream of the *H19* gene, and its insulator activity is involved in the imprinted expression of the distally-located *Igf2* gene (Supplementary Fig. 1a). On the maternal allele, the CTCF insulator protein bound to the unmethylated *H19* ICR sequence inhibits the action of the enhancers, located downstream of the *H19* gene, on the *Igf2* gene. On the other hand, in the paternally inherited allele, the absence of CTCF binding to the methylated *H19* ICR allows activation of *Igf2* by the enhancers^5–10^.

It is generally believed that regulation of the *H19* gene involves an epigenetic change at the *H19* promoter that is secondary to the methylation status of the *H19* ICR. On the paternal allele, both the *H19* ICR and the *H19* promoter are DNA methylated, and transcription of *H19* is repressed^11^. In mice lacking the paternal *H19* ICR, the methylation level of the *H19* promoter decreases, and *H19* is expressed^12^. In addition, when demethylation of the *H19* ICR was forcibly induced by epigenome editing in ES cells, the methylation level of the *H19* promoter decreased, and *H19* expression was activated in mice derived from these ES cells^13^. Thus, DNA methylation of the *H19* promoter seems mandatory for the repression of *H19* expression.

On the other hand, it has been reported that the paternal *H19* ICR represses *H19* expression via a mechanism independent of its promoter methylation status. Deletion of a 1.2 kb region of the paternal *H19* ICR resulted in derepression of the *H19* gene *in cis*, although methylation status of the rest of the *H19* ICR and of the *H19* promoter region remained unchanged^14^. Deletion of a ~0.9 kb region between CTCF binding sites 2 and 3 or mutation of the eight CpGs outside the CTCF binding sites of the paternal *H19* ICR also resulted in expression of *H19*, without altering the methylation status at the *H19* ICR and promoter^15^. These observations suggest that silencer activity exists in the paternal *H19* ICR or that the overall size or (methylated) CpG density is critical for conferring full repression activity on the paternal *H19* gene. Furthermore, Gebert et al. reported that knock-in of the *H19* ICR sequence at the mouse *Afp* locus repressed paternal *Afp* gene expression without altering the methylation level of the *Afp* gene promoter^16^.

As described above, *H19* gene expression appears to be regulated by multiple mechanisms (i.e., *H19* promoter methylation and the *H19* ICR itself). To date, however, no experiments have been able to directly determine whether methylation of the *H19* promoter is required for repression of the *H19* gene. This is largely because no experiments have been able to decrease *H19* promoter methylation without altering either the size or hypermethylation status of the *H19* ICR^12,13^.

Methylation of the paternal *H19* promoter changes dynamically during development. The *H19* locus, including the *H19* ICR, its downstream region, and the *H19* gene body, is highly and broadly methylated in sperm, with the exception of the *H19* promoter^5,6^. After fertilization and before implantation, the *H19* locus becomes extensively demethylated in the embryo, leaving only the *H19* ICR sequence methylated on the paternal allele. During postimplantation development, *de novo* DNA methylation occurs at the region downstream of the *H19* ICR through the *H19* promoter to the gene body^7,11,17^. Because this methylation requires the presence of a paternally hypermethylated *H19* ICR, the methylation seems to spread from the *H19* ICR in the downstream direction.

We previously generated transgenic mice (TgM) carrying a YAC transgene in which the mouse *H19* ICR fragment was inserted into the normally non-imprinted human beta-globin locus. Using these mice, we found that the Tg *H19* ICR was not methylated in sperm, but was *de novo* methylated after fertilization, only when paternally inherited^18^. This result demonstrated that the *H19* ICR has an intrinsic ability to acquire paternal allele-specific DNA methylation. In the establishment of post-fertilization imprinted methylation at the Tg *H19* ICR, the 5′ portion of the *H19* ICR was first methylated, and the 3′ portion became methylated later^19^. Furthermore, we identified a 118 bp sequence at the 5′ end of the *H19* ICR that was responsible for post-fertilization DNA methylation^20–22^.

Based on these observations, we assumed that methylation at the *H19* locus initiates from the 5′ portion of the *H19* ICR during the post-fertilization period in a 118 bp sequence-dependent manner, which then spreads further toward the *H19* promoter region during the post-implantation period. If this assumption is correct, we would predict that reversing the direction of the *H19* ICR would result in reduced methylation of the *H19* promoter region. This would allow us to investigate the relationship between methylation state of the *H19* promoter and the expression of the *H19* gene without altering the size of the *H19* ICR itself or its imprinted methylation status.

In this study, we generated mice in which the orientation of the *H19* ICR was inverted at the endogenous locus by genome editing. When the inverted ICR allele was paternally inherited, the methylation level of the *H19* promoter was reduced and the *H19* gene was derepressed, suggesting that methylation of the *H19* promoter itself is required for complete repression of *H19* expression. Unexpectedly, when the inverted allele was maternally inherited, the expression level of the *H19* gene was lower than that of the WT allele, even though the *H19* promoter remained fully hypomethylated. These observations, both expected and unexpected, should provide important insight into the mechanism of transcriptional regulation of imprinted genes in which polarity of the ICR plays a role within the locus.

## Results

### Generation of mutant mice carrying the *H19* ICR-inverted allele

To investigate the effect of *H19* ICR orientation on the imprinted expression and DNA methylation status of the *H19* locus, inversion of the *H19* ICR sequence was induced at the mouse endogenous locus by genome editing. To this end, gRNA and Cas9 protein were introduced into fertilized mouse eggs to cleave both sides of the ICR (Supplementary Fig. 1b). We also included single-stranded oligodeoxynucleotides (ssODNs) with sequences that correspond to the junctional sequences after correct inversion, expecting that this would facilitate the inverted ligation of the ICR fragment (Supplementary Fig. 1b). We introduced artificial restriction enzyme recognition sites into these ssODNs, allowing the use of the ssODNs in the inverted ligation reaction to be evaluated. Screening of F0 mice by PCR and DNA sequencing identified that although many of them carried a deletion between the two cleavage sites, three carried correctly inverted ICR alleles; two of those three carried the artificial enzyme sites (Supplementary Fig. 1c). We established mutant mouse lines (lines 21 and 23) from these two F0 mice.

### *H19* ICR inversion derepresses *H19* gene expression on the paternal allele

**Fig. 1:**
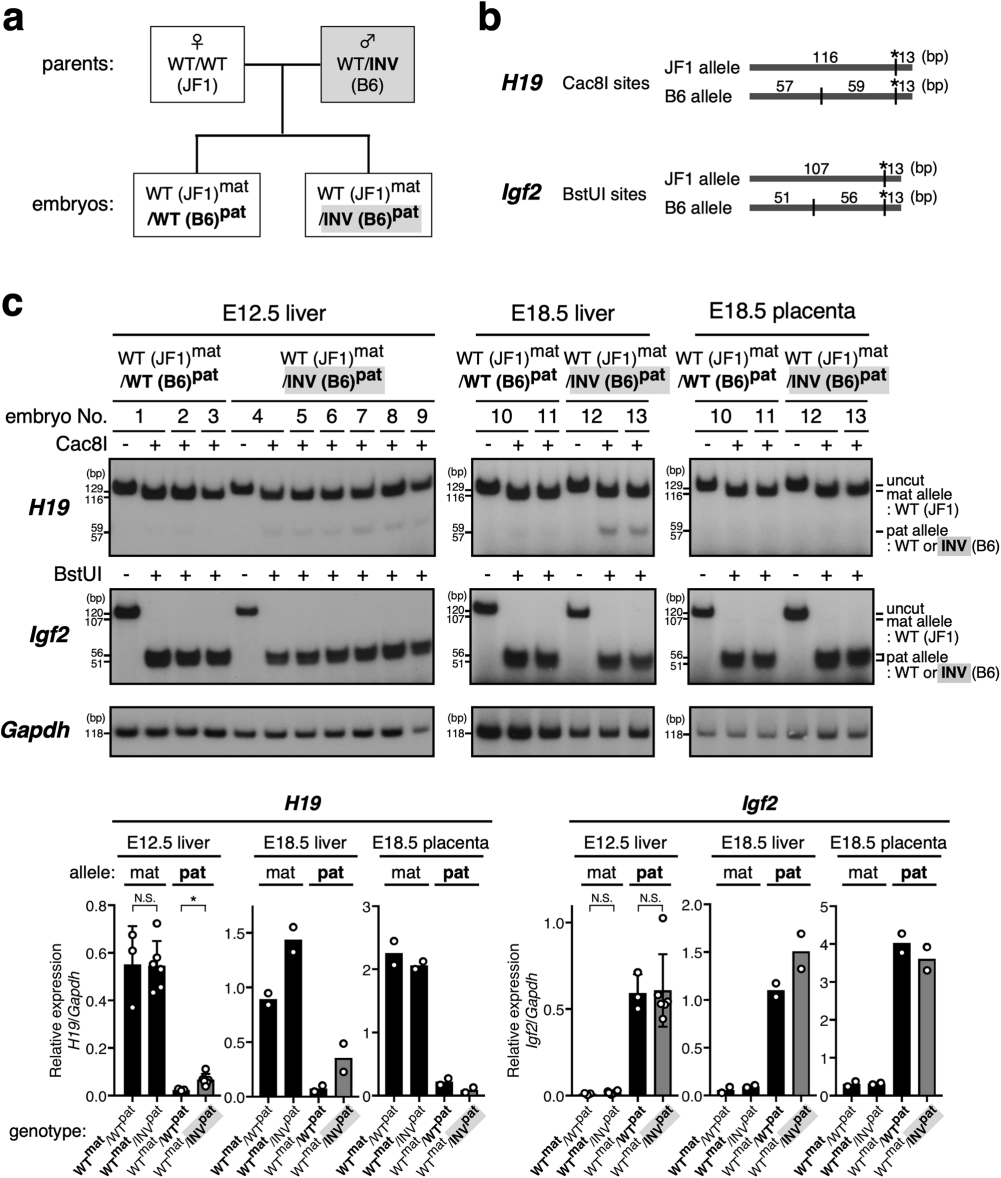
*H19* gene expression was derepressed on the paternally inherited inverted-ICR allele in fetal livers but not in placentas. **a** Breeding scheme. In order to distinguish parental origin of the alleles by using SNPs between inbred mouse strains, inverted-ICR heterozygous male mice (WT/INV; C57BL/6J [B6] background) were mated with wild-type female mice (WT/WT; JF1/Msf [JF1]), and embryos were obtained. **b**, **c** The allele-specific expression of the *Igf2* and *H19* genes was examined by RFLP analysis. Total RNA was extracted from livers (at E12.5 and E18.5) and placentas (E18.5), and *H19* and *Igf2* gene transcripts were amplified by RT-PCR followed by *Cac*8I or *Bst*UI digestions, respectively. Parental origin of transcripts was discriminated by allele-specific restriction sites. The asterisks in (**b**) indicate restriction sites introduced into primer sequence to monitor complete digestion of PCR products. The signal intensity of the bands was quantified and the ratio of *H19* or *Igf2* expression to that of *Gapdh* (arbitrary unit) was displayed on the graph. The means ± SD are shown for genes expression in E12.5 liver (*p < 0.05).

First, we examined the effect of altering the orientation of the *H19* ICR on expression of the imprinted gene in *cis* at the paternal allele. To distinguish the parental origin of the genes, we used single-nucleotide polymorphisms (SNPs) between mouse strains (C57B6/6J [B6] and JF1/Msf [JF1] background). To analyze *H19* and *Igf2* gene expression when the inverted ICR allele was inherited from the father, we crossed B6 males carrying the heterozygous mutant allele with wild-type JF1 females (Fig. 1a) and extracted RNA from embryonic tissues. RT-PCR products generated under quantitative amplification conditions were digested with restriction enzymes whose recognition sites are present only in the B6 allele (Fig. 1b). In the fetal liver (E12.5, E18.5), the paternal *H19* gene was repressed on the wild-type allele, while it was reproducibly expressed from the inverted ICR allele in two independent litters, although the expression level was much lower than from the maternal wild-type allele (Fig. 1c and Supplementary Fig. 2, *H19*). In the placenta (E18.5), expression of *H19* remained repressed on the inverted ICR allele, as it was on the wild-type allele. The *Igf2* genes were expressed on the inverted ICR, as were the wild-type alleles, only when they were paternally inherited (Fig. 1c and Supplementary Fig. 2, *Igf2*). These results indicated that ICR orientation did not affect *Igf2* gene activation on the paternal allele, but it did have an effect on the expression of *H19* in a tissue-specific manner.

### Effect of *H19* ICR inversion on methylation level inside the *H19* ICR

To determine whether derepression of paternal *H19* caused by the inversion of the *H19* ICR in fetal liver was due to a change in the DNA methylation status of the locus, we conducted bisulfite sequencing analysis of tissue samples. First, we analyzed the inside of the inverted ICR and found that it was highly methylated in both liver (E12.5 and E18.5) and placenta (E18.5), as in the wild-type ICR (Supplementary Fig. 3, regions I and II in WT, and regions III and IV in inverted ICR). Internal methylation of the ICR was stably maintained throughout development, and postnatal tail DNA was also highly methylated at the paternal allele (Supplementary Fig. 4). In addition, the methylation state was reprogrammed across generations and was consistent between two independent strains (Supplementary Fig. 4). Consistent with our previous observation that the *H19* ICR has the intrinsic ability to establish and maintain imprinted DNA methylation after fertilization^18,19,23^, the inside of the *H19* ICR was properly methylated regardless of its orientation within the locus.

### Effect of *H19* ICR inversion on methylation levels of the paternally inherited *H19* locus

**Fig. 2:**
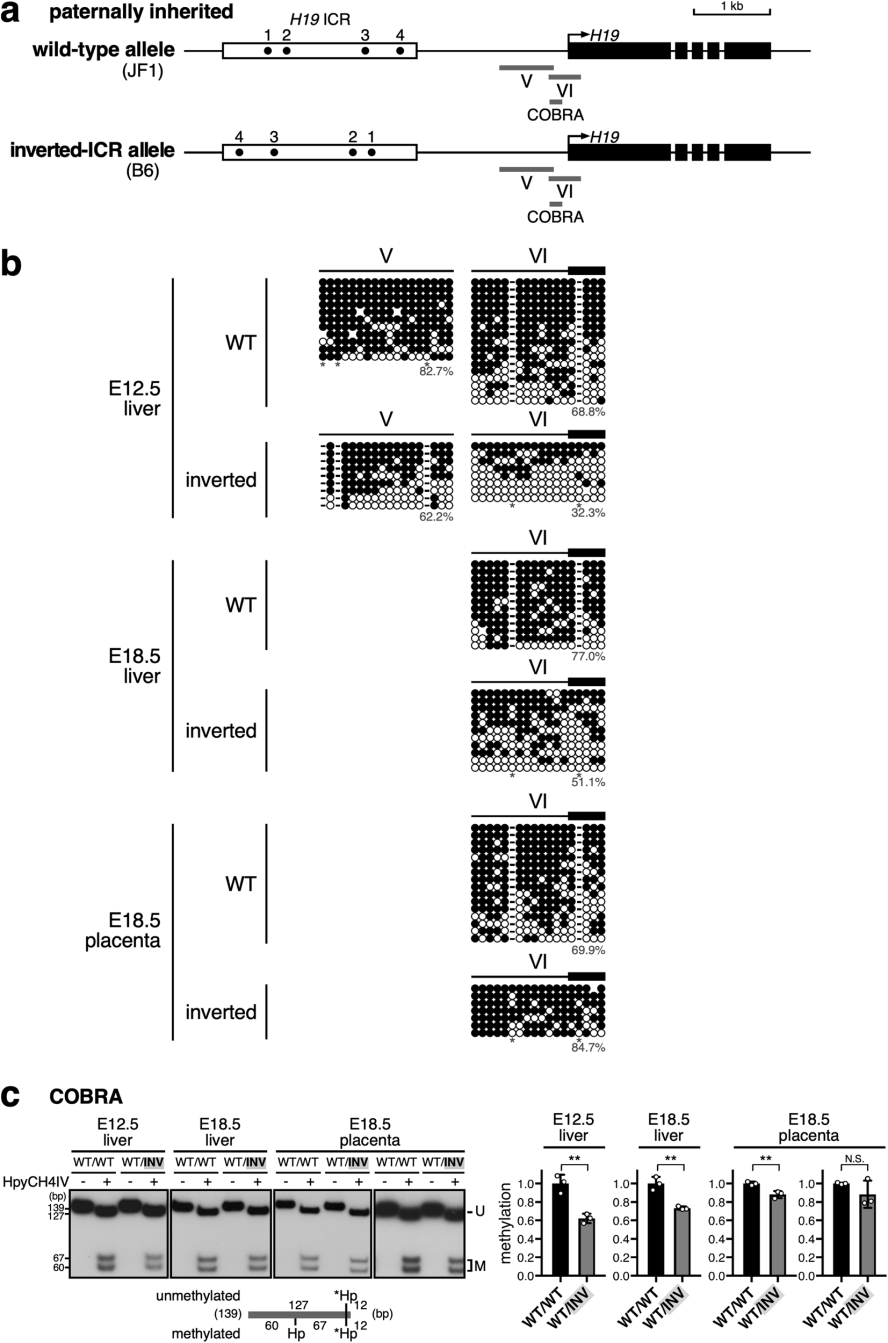
DNA methylation levels of the *H19* promoter was decreased on the paternally inherited inverted-*H19* ICR allele in fetal livers but not in placentas. **a** Map of wild-type and inverted-ICR alleles. Regions analyzed by bisulfite sequencing in (**b**) and COBRA in (**c**) were indicated by gray bars below the map. **b**, **c** DNA methylation status of the *H19* promoter on the paternally inherited wild-type and inverted *H19* ICR alleles in livers (E12.5 and E18.5) and placentas (E18.5). **b** Bisulfite sequencing. For analyses of paternally inherited wild-type (WT) allele, genomic DNA of tissues from INV(B6)^mat^/WT(JF1)^pat^ embryos, which were identical to ones analyzed in Fig. 3c (E12.5, No. 5−7; E18.5, No. 13−16) were pooled. For analyses of paternally inherited inverted-ICR allele, genomic DNA of tissues from WT(JF1)^mat^/INV(B6)^pat^ embryos, which were identical to ones in Fig. 1c (E12.5, No. 4−6; E18.5, No.12−13) were pooled. The parental origin of the alleles was determined by SNPs between B6 and JF1. Each horizontal row represents a single DNA template molecule. Methylated and unmethylated CpG motifs are shown as filled and open circles, respectively. The methylation levels (%) of CpGs excluding the allele-specific sites (*) are shown for each cluster and were statistically compared (WT vs inverted at region V, *p* = 0.1860 [E12.5 liver], region VI, *p* = 0.0174 [E12.5 liver], *p* = 0.0187 [E18.5 liver], *p* = 0.1566 [E18.5 placenta]). **c** COBRA. Genomic DNA of tissues from embryos which were analyzed in Fig. 1c or Supplementary Fig. 2 was pooled according to their genotypes (E12.5 WT/WT, No. 1−3, WT/INV, No. 4−6; E18.5 liver and placenta (left) WT/WT, No. 10−11, WT/INV, No. 12−13 in Fig. 1c; E18.5 placenta (right) WT/WT, No. 21-22, WT/INV, No. 23−24 in Supplementary Fig. 2). The PCR products were digested with *Hpy*CH4IV (Hp) (+) to assess the methylation status of the original DNA. The asterisks indicate restriction sites introduced into primer sequence to monitor the complete digestion of PCR products. The signal intensity of the bands was quantified and the ratio of the intensity of the digested fragments (i.e., methylated, M) to that of the overall fragments (U + M) was calculated, as the value of WT/WT set as 1. The mean ± SD for three technical replicates was displayed on the graph (**p < 0.01).

Next, we investigated whether derepression of *H19* gene was due to a change in the DNA methylation status of the *H19* promoter. In the liver (E12.5, E18.5), where *H19* expression was derepressed, the methylation level of the *H19* promoter (regions V, VI, and COBRA in Fig. 2a) was lower in the inverted ICR allele than in the wild-type allele in two independent litters (Fig. 2b, c and Supplementary Fig. 5). This observation indicated that inverting the orientation of the ICR caused a modest loss of DNA methylation at the *H19* promoter, even though the *H19* ICR itself was correctly methylated. On the other hand, in placenta, the *H19* promoter remained properly methylated in the inverted ICR allele (Fig. 2b, c and Supplementary Fig. 5). Thus, reduced methylation of the *H19* promoter coincided with derepression of the paternally derived *H19* gene, suggesting that methylation of the *H19* promoter is required for complete repression of the *H19* gene.

We then looked for regions other than the *H19* promoter where DNA methylation was altered by reversal of the *H19* ICR. In both liver and placenta, although the methylation level of the upstream region tended to be higher in the inverted ICR than in the wild-type alleles (regions VII and XI in WT and inverted ICR alleles, respectively, in Supplementary Figs. 6 and 7), differences in methylation levels between wild-type and inverted alleles were not statistically significant. Immediate downstream region of the ICR was highly and equally methylated in the inverted ICR, as well as in the wild-type alleles, in both liver and placenta (regions VIII and XII in WT and inverted ICR alleles, respectively, in Supplementary Fig. 6). Even if the activity of the *H19* ICR involved in establishing DNA methylation has polarity, it seems capable of introducing DNA methylation at the immediate vicinity in both directions. Based on reports that methylation of the *H19* gene body may regulate expression of the *H19* gene^24^, we also compared its methylation status between the wild-type and inverted ICR alleles in both liver and placenta, but found no significant differences (regions IX and X in Supplementary Fig. 6). Thus, the methylation status of these regions (immediate downstream of the *H19* ICR and *H19* gene body) does not appear to regulate paternal *H19* gene expression because it was consistent between (wild-type and inverted ICR) alleles and tissues.

Based on these observations, we infer that DNA methylation of the paternally inherited *H19* gene promoter is regulated by orientation-dependent *H19* ICR activity, and that promoter hypermethylation is required for complete repression of the paternal *H19* gene.

### *H19* ICR inversion represses the *H19* gene expression on the maternal allele

**Fig. 3:**
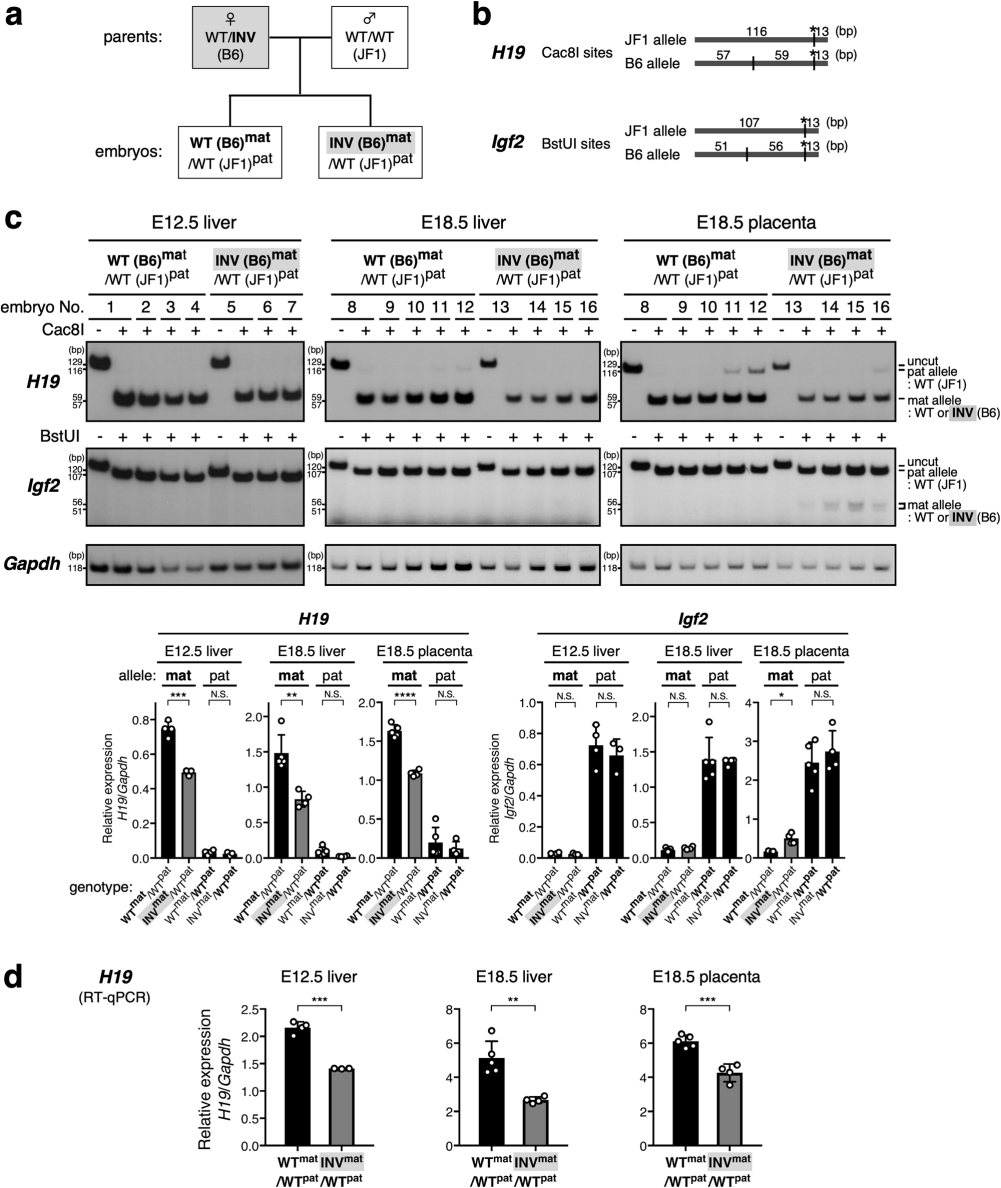
*H19* gene expression was repressed on the maternally inherited inverted-ICR allele in fetal tissues. **a** Breeding scheme. In order to distinguish parental origin of the alleles by using SNPs between inbred mouse strains, inverted-ICR heterozygous female mice (WT/INV; B6 background) were mated with wild-type male mice (WT/WT; JF1) to obtain embryos. **b**, **c** The allele-specific expression of the *Igf2* and *H19* genes was examined by RFLP analysis. Total RNA was extracted from livers (at E12.5 and E18.5) and placentas (E18.5), and *H19* and *Igf2* gene transcripts were amplified by RT-PCR followed by *Cac*8I or *Bst*UI digestions, respectively. Parental origin of transcripts was discriminated by allele-specific restriction sites. The asterisks in (**b**) indicate restriction sites introduced into primer sequence to monitor complete digestion of PCR products. The signal intensity of the bands was quantified and the ratio of *H19* or *Igf2* expression to that of *Gapdh* was calculated (arbitrary unit) and the means ± SD was displayed on the graph (*p < 0.05, **p < 0.01, ***p < 0.001, ****p < 0.0001). **d** The expression levels of the *H19* gene in the samples analyzed in (**c**) were also examined by RT-qPCR. The ratio of *H19* expression to that of *Gapdh* was calculated (arbitrary unit) and the means ± SD are shown (**p < 0.01, ***p < 0.001).

**Fig. 4:**
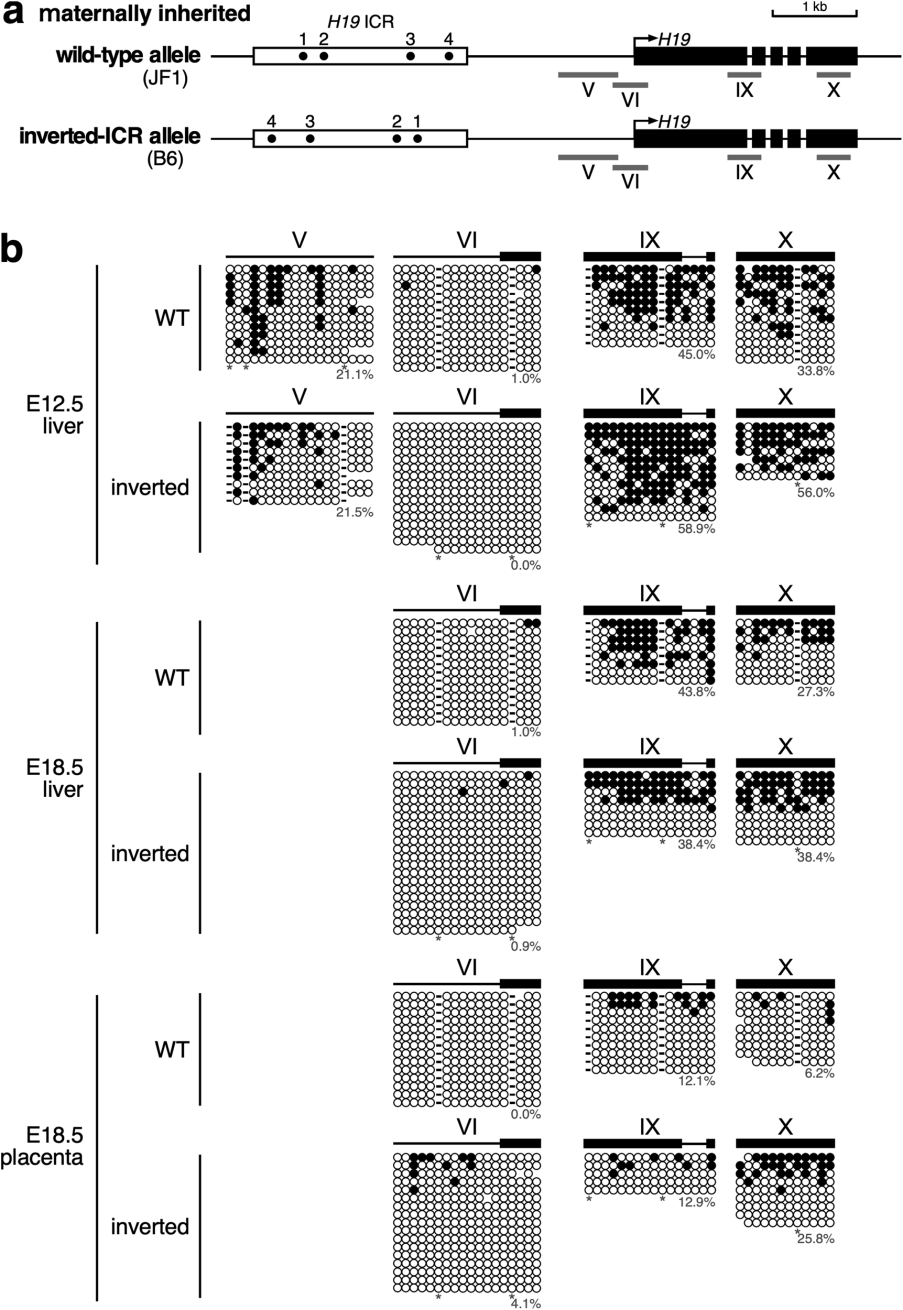
Methylation status of the maternally inherited *H19* locus in fetal tissues. **a** Map of wild-type and inverted-ICR alleles. Regions indicated by gray bars below the map were analyzed by bisulfite sequencing in (**b**). **b** For analyses of maternally inherited wild-type (WT) allele, genomic DNA of tissues from WT(JF1)^mat^/INV(B6)^pat^ embryos which were identical to ones analyzed in Fig. 1c (E12.5, No. 4−6; E18.5, No. 12−13) were pooled. For analyses of maternally inherited inverted allele, genomic DNA of tissues from INV(B6)^mat^/WT(JF1)^pat^ embryos which were identical to ones in Fig. 3c (E12.5, No. 5−7; E18.5, No.13−16) were pooled. The parental origin of the alleles was determined by SNPs between B6 and JF1. Each horizontal row represents a single DNA template molecule. Methylated and unmethylated CpG motifs are shown as filled and open circles, respectively. The methylation levels (%) of CpGs excluding the allele-specific sites (*) are shown for each cluster. There was no significant difference in methylation levels between wild-type and inverted alleles, except for region VI in E18.5 placenta (*p* = 0.0300).

We next examined the effect of inverting the *H19* ICR on the maternal allele (Fig. 3a). Allele-discriminating gene expression analysis using SNPs (Fig. 3b) revealed that the expression level of the *H19* gene from the inverted ICR allele was lower than that from the wild-type allele in both liver (E.12.5, 18.5) and placenta (E18.5) in two independent litters (Fig. 3c and Supplementary Fig. 8a); this result was confirmed by RT-qPCR (Fig. 3d and Supplementary Fig. 8b). We then analyzed the DNA methylation status of the *H19* promoter and found that the promoter was hypomethylated on both the inverted and wild-type alleles (regions V and VI in Fig. 4 and Supplementary Fig. 9). The methylation level of the *H19* gene body also did not differ between wild-type and inverted ICR alleles (regions IX and X in Fig. 4). Therefore, the orientation of the *H19* ICR does not affect the methylation status of the *H19* promoter or gene body on the maternal allele, and the methylation statuses of these regions are not likely to regulate the *H19* expression on the maternal allele.

### Effect of *H19* ICR inversion on methylation levels of the maternally inherited *H19* locus

We then conducted methylation analysis of inside of the *H19* ICR and found that the sequence was hypomethylated in the inverted ICR, as well as in the wild-type alleles, in both liver and placenta. However, the extent of the hypomethylated region in the inverted ICR was smaller than in the wild-type ICR (regions I/II and III/IV in the WT and inverted ICR, respectively, in Supplementary Fig. 10). In addition, the methylation level in regions immediately downstream of the ICR was significantly higher in the inverted ICR allele than in the wild-type allele (regions VIII (WT) and XII (inverted) in Supplementary Figs. 10 and 11). Because this ectopic DNA methylation occurred in both liver and placenta, we hypothesized that it is responsible for the reduction in *H19* expression from the maternally inherited inverted ICR allele. In other words, the downstream region of the *H19* ICR may be involved in transcriptional regulation of the *H19* gene in a DNA methylation-dependent manner. Such an activity has not been previously reported in this region of the locus.

### Search for a transcriptional regulatory activity around the *H19* downstream region

We then hypothesized that the activity of the previously unidentified *H19* enhancer is located downstream of the ICR and becomes attenuated when it is methylated by ICR inversion, resulting in reduced *H19* transcription. To test this hypothesis in cell transfection assays, we cloned the *H19* ICR downstream sequence (ICR-DS in Supplementary Fig. 12) and linked it to *H19* or SV40 promoters on luciferase reporter plasmids. However, the ICR-DS fragment did not show any transcriptional activity in transiently transfected MEF cells (Supplementary Fig. 12). Therefore, it seems unlikely that a transcriptional regulatory element in this region acquires DNA methylation in the maternally inherited inverted ICR allele and causes downregulation of *H19* gene expression. This suggested that in the maternal allele, the direction of the *H19* ICR affects the transcription of the *H19* gene through a mechanism that is independent of DNA methylation.

## Discussion

*H19* gene encodes a long noncoding (lnc) RNA that is highly expressed in developing embryo, and then repressed immediately after birth except in muscle^25^. Because *H19* lncRNA and its processed product, miR-675, are associated with suppression of fetal/placental growth and cell cycle regulation, abnormal expression of these RNAs may cause tumorigenesis and other diseases^26–29^. Thus, elucidation of the mechanism underlying transcriptional regulation of *H19* is fundamental to clarifying the causes of these diseases and to develop new therapies for treatment.

In this study, we found that inversion of the paternally inherited *H19* ICR decreased the methylation level of the *H19* promoter and that the paternal *H19* gene was concurrently expressed in fetal liver, while the *H19* ICR itself remained properly methylated. Although the transcriptional expression level of a gene and its DNA methylation status affect each other, we believe that the reduced methylation of the *H19* gene promoter in our mutant mice was not the consequence of ectopic *H19* gene activation in the paternal inverted ICR allele. In previously reported mice with partial deletion of the *H19* ICR sequence, the paternal *H19* gene was derepressed (presumably due to a loss of transcriptional repressive activity of the *H19* ICR), despite the fact that the *H19* promoter in *cis* remained correctly methylated^14,15^, i.e., *H19* transcription does not necessarily cause demethylation of the promoter. We, therefore, assume that the paternal *H19* gene was derepressed as a result of reduced methylation of the *H19* promoter caused by inversion of the orientation of the *H19* ICR. In other words, hypermethylation of the *H19* promoter is necessary for complete repression of the *H19* gene. Taking these observations together, the repression of the paternal *H19* gene seems to involve multiple mechanisms: the transcriptional repressive activity of the *H19* ICR itself and the methylation of the *H19* promoter. Formally, we cannot rule out the possibility that inversion of the *H19* ICR placed the hypothetical silencer element too far away from the *H19* gene in our mutant mice, resulting in derepression of the *H19* gene independent of its promoter methylation status. In accordance with this notion, Gebert et al. reported that knock-in of the *H19* ICR sequence at the paternal *Afp* locus repressed the gene even without changing promoter methylation^16^; however, this result may not be applicable here, as the *Afp* promoter contains far fewer CpGs and the distance to the *H19* ICR is much shorter.

The effect of the *H19* ICR inversion on *H19* promoter methylation and gene expression in the paternal allele was tissue-specific, i.e., it was observed in liver but not in placenta. Epigenetic regulation at multiple imprinted loci differs between extraembryonic and embryonic tissues^30–33^, suggesting the existence of tissue-specific regulatory factors. The *H19* locus may also be regulated by these factors.

On the other hand, *H19* expression level was reduced on the maternally inherited inverted ICR allele, even though the *H19* promoter remained hypomethylated, as on the WT allele. We, therefore, assumed that the DNA methylation status of transcriptional regulatory sequences other than the *H19* promoter may have been altered on the maternal allele, resulting in *H19* expression. Although we found a candidate sequence that was significantly more methylated on the maternal inverted ICR allele than on the WT allele immediately downstream of the *H19* ICR, reporter assays revealed no transcriptional regulatory activity in this sequence. The maternally inherited *H19* ICR sequence itself is required for full activation of the *H19* gene^34^, and CTCF binding sites in the *H19* ICR are essential for the formation of maternal allele-specific chromatin modification status and appropriate interaction between the enhancer and *H19* promoter^35–37^. These processes may be somehow impaired by the inversion of the *H19* ICR.

Although inversion of the *H19* ICR altered the expression levels of *H19* in both parental alleles, it had no obvious effect on the weights of either embryos or placentas (Supplementary Fig. 13). We, therefore, concluded that the change in *H19* expression level induced by the *H19* ICR inversion was not sufficient to affect embryonic growth.

In contrast to *H19*, the expression of *Igf2* was not altered by inversion of the *H19* ICR. The mouse *H19* ICR contains four CTCF/cohesin-binding motifs^8,9,38^, and the binding of the complexes to sites on hypomethylated maternal *H19* ICR represses *Igf2* expression via its insulator activity. Insulator activity arises from the functional isolation of specific genomic regions by chromatin loops formed by association of two CTCF/cohesin sites^39^; the orientation of the CTCF binding sequence is normally in opposite directions and regulates the mode of loop formation. In the *Pcdh* and *β-globin* loci, reversing the orientation of one of the CTCF sites in either the promoter or enhancer region changed the topology of the loops and hence the pattern of gene expression^40^. Recent work showed that the maternal *H19* ICR formed loop structures with several downstream CTCF clusters, each of which contains CTCF binding sites in the opposite direction to the ones within the *H19* ICR^41^. The expression of maternally inherited *Igf2* was not affected on the inverted ICR allele in our study, implying that the spatial structure of the locus may have a trivial effect on *Igf2* gene regulation on the maternal allele or that the gross structure of the gene locus is not altered by inversion of the entire *H19* ICR. It would be interesting to investigate whether the *H19* ICR inversion altered the spatial structure of the locus. Curiously, slight derepression of *Igf2* was observed on the inverted maternal allele in placenta (Fig. 3c and Supplementary Fig. 8a). This may be due to the unusual increase in methylation inside the ICR (E18.5 placenta, Supplementary Fig. 10b) and reduced binding to CTCF insulator protein, which then allows interaction between the enhancer and *Igf2* promoter.

In this study, we hypothesized that *H19* promoter methylation on the paternal allele is a result of methylation spreading downwards from the 118-bp sequence of the *H19* ICR. Based on this, we analyzed the effect of inverting the *H19* ICR on genomic imprinting in mice. Although the methylation level of the paternal *H19* promoter was decreased as expected, the spreading of methylation, if any, may not have been rigorously unidirectional, as the region between the ICR and the *H19* promoter remained highly methylated. Generally, it is accepted that the presence of transcription and the H3K36me histone mark in oocytes and sperm are prerequisite for *de novo* DNA methylation during gametogenesis^42–44^. It remains to be fully elucidated where *de novo* DNA methylation initiates in somatic cells (i.e., is there a DNA methylation origin that recruits methyltransferases?) and how DNA methylation states propagate to the surrounding regions. Our mutant mice may help to obtain insights into such molecular mechanisms and to understand the etiology of diseases involving DNA methylation abnormalities, such as genomic imprinting disorders and oncogenesis.

## Methods

### Mice

Mice were housed in a pathogen-free barrier facility in a 12 h light/12 h dark cycle, and fed standard rodent chow. Animal experiments were performed in a humane manner and approved by the Institutional Animal Experiment Committee of the University of Tsukuba. Experiments were conducted in accordance with the Regulation of Animal Experiments of the University of Tsukuba and the Fundamental Guidelines for Proper Conduct of Animal Experiments and Related Activities in Academic Research Institutions under the jurisdiction of the Ministry of Education, Culture, Sports, Science and Technology, Japan.

### Generation of “inverted-ICR” mice by CRISPR/Cas9 genome editing

The oligonucleotides were annealed, phosphorylated, and ligated to the *Bbs*I site of pX330 (plasmid 42230; Addgene)^45^ for generating Cas9/sgRNA expression vectors. In the following sequences, overhanging nucleotides are shown in lowercase letters. The sequences for the 5′ border are 5′-caccGGCAGTAAGCTTTGGCGGGG-3′ and 5′-aaacCCCCGCCAAAGCTTACTGCC-3′; for the 3′ border they are 5′-caccGTTTCGGTGGACGCACGCACG-3′ and 5′-aaacCGTGCGTGCGTCCACCGAAAC-3′. We used ssODNs (IDT, Coralville, IA, USA) with sequences that correspond to the junctional sequences after correct inversion. The sequence of ssODN for 5′ junction is 5′-ctgtttgcccaccagctgctagccatcacctagtcctcaatgtcacgtactattacaatggccaaaacagactagacttgaccccaagagcccccctcgagcgcggcagtttctatgtctcccgcctataaccgattctgtattgagtttggattgaacagatctggctagcttgaggagtcccaaggcagaaggggacc-3′, and for 3′ junction that is 5′-atagagctagatctcttcttccagaaacaagttaggcatgcctttgtcaatctggggactgccagggcagaaagtacaatgagggcagtaagctttggatccgtgcgtccaccgaaaccccatagccataaaagcagaggctggggttcaaccattgcaatgtcccaggtaacctaggaactgtagcaagaagttgcaaa-3′ (artificially introduced *Xho*I and *Bam*HI sites are underlined, respectively). The plasmids and ssODNs were microinjected into the pronuclei of fertilized eggs of C57BL/6J mice (Charles River Laboratories Japan, Kanagawa, Japan). Tail DNA from 27 founder offspring was screened by PCR and sequencing.

### Preparation of embryos

Inverted-ICR mice (which was generated by using C57BL/6J strain, and has a genetic background of *Mus musculus domesticus*) were mated with wild-type JF1/Msf^46^ mice (which was provided by the RIKEN BRC through the National Bio-Resource Project of the MEXT, Japan, and of which genome is basically from *Mus musculus molossinus*) to distinguish the parental origin of the alleles in the offspring. Livers are obtained from E12.5 and E18.5 embryos, and placentas are recovered at E18.5. Each tissue was divided into two and used for the preparation of total RNA and genomic DNA, respectively.

### Allele-specific expression analysis using restriction fragment length polymorphism (RFLP)

Inverted-ICR mice (C57BL/6J) carrying the heterozygous mutant allele were crossed with wild-type JF1/Msf mice. Total RNA was recovered from livers and placentas of embryos using ISOGEN (Nippon Gene, Tokyo, Japan) and converted to cDNA using ReverTra Ace qPCR RT Master Mix with gDNA Remover (TOYOBO, Osaka, Japan). PCR was performed using AmpliTaq Gold 360 Master Mix (Thermo Fisher Scientific, Waltham, MA, USA) and PCR primers listed in Supplementary Table 1 with α-^32^P-dCTP (NEG513H, PerkinElmer, Waltham, MA, USA) at quantitative amplification condition (94 °C for 3 min, followed by 21 cycles of 94 °C for 30 s, 58 °C for 30 s, and 72 °C for 1 min). The amplified products were digested with *Cac*8I or *Bst*UI, in order to discriminate the parental origin of the transcripts, and subjected to polyacrylamide gel electrophoresis. The restriction sites were also introduced into primer sequences so that the complete digestion of PCR products can be concomitantly monitored. Gels were dried, subjected to phosphorimaging on a Typhoon 8600 (GE healthcare, Chicago, IL, USA), and then subjected to quantitative analysis using ImageQuant. X-ray autoradiography was also performed. Uncropped and unedited images are shown in Supplementary Figs. 14−16.

### RT-qPCR

Total RNA from livers or placentas of E12.5 or E18.5 embryos was converted to cDNA as described above. Quantitative amplification of cDNA was performed with the Thermal Cycler Dice (TaKaRa Bio, Shiga, Japan) using TB Green Premix EX TaqII (TaKaRa Bio). PCR primers are listed in Supplementary Table 1.

### DNA methylation analysis by bisulfite sequencing

For DNA methylation analysis of paternally inherited inverted-ICR allele and maternally inherited wild-type allele, inverted-ICR male mice (C57BL/6J background, carrying the heterozygous mutant allele) and wild-type female mice (JF1/Msf) were mated and heterozygous mutant embryos were obtained. Conversely, for analysis of maternally inherited inverted-ICR allele and paternally inherited wild-type allele, heterozygous inverted-ICR females and wild-type males were mated and heterozygous mutant embryos were obtained. Genomic DNA extracted from tissues of embryos was pooled as described in Figure legends and digested with *Xba*I, and then treated with sodium bisulfite using the EZ DNA Methylation Kit (Zymo Research, Irvine, CA, USA) by following the manufacturer’s instruction. Subregions of the wild-type or inverted *H19* ICR alleles were amplified by PCR using EpiTaq HS (TaKaRa Bio). The PCR products were subcloned into the pGEM-T Easy vector (Promega, Madison, WI, USA) for sequencing analyses. PCR primers are listed in Supplementary Tables 2 and 3. Sequencing results were analyzed by using Quantification tool for Methylation Analysis (QUMA, http://quma.cdb.riken.jp). We checked whether each C that is not present in the CpG motif was correctly converted to T, and used only results from clones with a CT conversion efficiency of 95% or higher.

### DNA methylation analysis by Southern blotting

Genomic DNA extracted from tail tips of ~1-week-old animals was first digested by *Nhe*I and *Sac*I, and then subjected to the methylation-sensitive enzyme *Bst*UI. Following size separation in agarose gels, Southern blots were hybridized with α-^32^P-labeled probes and subjected to X-ray film autoradiography. Uncropped and unedited images are shown in Supplementary Fig. 15.

### Combined bisulfite restriction analysis (COBRA)

Genomic DNA was pooled as described in Figure legends and treated with sodium bisulfite. PCR was performed using EpiTaq HS (TaKaRa Bio) and primers listed in Supplementary Tables 2 and 3 with α-^32^P-dCTP (Perkinelmer) under quantitative amplification condition (94 °C for 5 min, followed by 28 cycles of 94 °C for 1 min, 60 °C for 2 min, and 72 °C for 2 min). After purification with Autoseq G-50 columns (Cytiva, Tokyo, Japan), the aliquots of PCR products were digested with *Hpy*CH4IV enzyme and subjected to polyacrylamide gel electrophoresis. The restriction sites were also introduced into primer sequence so that complete digestion of PCR products can be concomitantly monitored. Gels were dried, subjected to phosphorimaging on a Typhoon 8600, and then subjected to quantitative analysis using ImageQuant. X-ray autoradiography was also performed. Uncropped and unedited images are shown in Supplementary Fig. 14.

### Reporter plasmid construction

Reporter plasmids were generated based on a PGV-B2 or PGV-P2 vectors (Toyo Ink, Tokyo, Japan). The *H19* promoter and ICR-DS plus *H19* promoter sequences were PCR amplified using the following primer sets and a template plasmid in which the *H19* gene locus sequence is subcloned: 5′- TCGGCCTGTCGACTGCTGATGCTG-3′ and 5′- ACCCCCCCTCGAGCTCCCACACC-3′ (*Sal*I and *Xho*I sites are underlined, respectively) or 5′- AACTGCCTCGAGCGTGCGTC-3′ and 5′- ACCCCCCCTCGAGCTCCCACACC-3′ (*Xho*I sites are underlined). The products were digested with appropriate enzymes, and ligated to the *Xho*I site of PGV-B2 to generate PGV-B2/H19pr or PGV-B2/ICRDS-H19pr plasmids, respectively. The ICR-DS sequence was PCR-amplified using the following primer set: 5′- AACTGCCTCGAGCGTGCGTC-3′ and 5′- AGCATCAGTCGACTAAAGGCCGAG-3′ (*Xho*I and *Sal*I sites are underlined, respectively). The product, digested with *Xho*I and *Sal*I, was ligated to the *Xho*I site of PGV-P2 to generate PGV-P2/ICRDS plasmid.

### Cell culture and transfection

Mouse embryonic fibroblasts (MEF) cells, generated from JF1/C57BL6J F1 embryo (E13.5), were maintained in Dulbecco’s modified Eagle’s medium (08458-45, nacalai tesque, Kyoto, Japan) containing 10% fetal bovine serum (Biological Industries, Kibbutz Beit-Haemek, Israel) and penicillin-streptomycin (Sigma, St. Louis, MO, USA) on gelatin-coated dishes. For luciferase assays, MEF cells were seeded in gelatin-coated 24-well plates at a density of 2 × 10^4^ cells/well 12 h prior to transfection. Equimolar amounts of test reporter plasmid, 25 ng of pCMV-β-Gal (where CMV is cytomegalovirus and β-Gal is β-galactosidase), and pUC19 (to make a total plasmid weight of 275 ng) were introduced by using 0.75 µl of Gene Juice transfection reagent (Millipore, Burlington, MA, USA). Forty-eight hours after transfection, cells were harvested, and luciferase and β-Gal activities (for the correction of variation in transfection efficiencies) were measured by using a Centro XS3 LB 960 Microplate Luminometer (Berthold Technologies, Bad Wilbad, Germany) and X-Mark spectrophotometer (Bio-Rad, Hercules, CA, USA), respectively.

### Statistics and reproducibility

Statistical analysis was performed by two-tailed unpaired Student’s t-test and Welch’s t-test using Prism 7 (GraphPad Software). The means and standard deviation were indicated. In bisulfite sequencing analysis, Mann−Whitney U-test was performed using QUMA. *p* values < 0.05 were considered significant. The sample numbers were described in each figure legend.

### Reporting summary

Further information on research design is available in the Nature Research Reporting Summary linked to this article.

## Supplementary information

Supplementary Information

Description of Additional Supplementary Files

Supplementary Data 1

Reporting Summary

## Data Availability

Source data for the graphs are available in Supplementary Data 1. Sanger sequencing data for bisulfite sequencing analysis are available from Dryad (10.5061/dryad.wdbrv15qb)^47^. Any remaining information can be obtained from the corresponding author upon reasonable request.

## Code availability

The data were plotted graphically by using Prism 7 (GraphPad Software). No custom code was used in this study.
