# Supplementary material for: Orientation of mouse H19 ICR affects imprinted H19 gene expression through promoter methylation-dependent and -independent mechanisms
Source: Commun Biol. 2021 Dec 17;4:1410. doi: 10.1038/s42003-021-02939-9 (PMC8683476; doi:10.1038/s42003-021-02939-9)
Supplement: Supplementary file 1 — Supplementary Information [file 42003_2021_2939_MOESM1_ESM.pdf]

Orientation of mouse *H19* ICR affects imprinted *H19* gene expression through promoter methylation–dependent and –independent mechanisms

Hitomi Matsuzaki[1, 2, *], Yu Miyajima[3], Akiyoshi Fukamizu[2], Keiji Tanimoto[1, 2]

[1] Faculty of Life and Environmental Sciences, University of Tsukuba, Tsukuba, Ibaraki, Japan

[2] Life Science Center for Survival Dynamics, Tsukuba Advanced Research Alliance (TARA), University of Tsukuba, Tsukuba, Ibaraki, Japan

[3] Graduate school of Life and Environmental Sciences, University of Tsukuba, Tsukuba, Ibaraki, Japan

[*] To whom corresponding should be addressed. E-mail: matsuzaki@tara.tsukuba.ac.jp

Supplementary Information

Supplementary Tables 1-3

Supplementary Figures 1-16

Supplementary Table 1. Primer sequences for allele-specific expression analysis and RT-qPCR.

gene		name	sequence
H19	forward	mH19-5S2	5'- CGGTGTGATGGAGAGGACAGAAG -3'
	reverse	mH19-3A2	5'- CCAGAGAGCAGCAGGCAAGTGTTAG -3'
Igf2	forward	mIgf2-5S2	5'- TCTGTGCGGAGGGGAGCTTGTT -3'
	reverse	mIgf2-3A2	5'- CAGCACTCTTCCGCGATGCCAC -3'
GAPDH	forward	mGAPDH-5S	5'- AAAATGGTGAAGGTCGGTGTG -3'
	reverse	mGAPDH-3A	5'- TGAGGTCAATGAAGGGGTCGT -3'

Supplementary Table 2. Primer sets for bisulfite sequencing analysis and COBRA.

region analyzed	forward	reverse
I	ICR-MA-5S11	ICR-MA-3A27
II	ICR-MA-5S20	ICR-MA-3A6
III	ICRas-MA-5S1	ICR-MA-3A6
IV	ICR-MA-5S11	ICRas-MA-3A1
V	H19-MA-5S5	H19-MA-3A4
VI	H19-MA-5S8	H19-MA-3A6
VII	ICR-MA-5S24	ICR-MA-3A30
VIII	ICR-MA-5S22	ICR-MA-3A28
IX	H19as-MA-5S1	H19as-MA-3A1
X	H19as-MA-5S2	H19as-MA-3A2
XI	ICRas-MA-5S3	ICR-MA-3A30
XII	ICR-MA-5S22	ICRas-MA-3A3
COBRA	H19as-MA-5S3	H19as-MA-3A3

Supplementary Table 3. Primer sequences for bisulfite sequencing analysis and COBRA.

Name	sequences
ICR-MA-5S11	5'-TTTGAGGTATTGAATTTGGGTGAT-3'
ICR-MA-5S20	5'-GGGATATTGTAATGGTTGAATTTT-3'
ICR-MA-5S22	5'-ATTTTTTTTATGGTTTTTATATTTTTTG-3'
ICR-MA-5S24	5'-AAGTTTTTTTGGTTAGGTTAAATT-3'
ICRas-MA-5S1	5'-AGATTAGATTTGATTTTAAGAGTT-3'
ICRas-MA-5S3	5'-TTGGGATTTTTTAAGTTAGTTAGA-3'
H19-MA-5S5	5'-ATTTTTATTTTGGAATTTTTTAGATAGG-3'
H19-MA-5S8	5'-TTGAGTTAGTTTTTTAGTTTTTTAATAT-3'
H19as-MA-5S1	5'-TATTTTTAGGGGAGTTAAGGGTATAGGA-3'
H19as-MA-5S2	5'-TTTTTGAGAATTTATTTTTATGGTTAAT-3'
H19as-MA-5S3	5'-GTTGTGGTGAGGTTGTTTTTGGAG-3'
ICR-MA-3A6	5'-ATATACACCTCTAAAATAATTCCC-3'
ICR-MA-3A27	5'-CAAAACAAACTAAACTTAACCCC-3'
ICR-MA-3A28	5'-CTATTCAATCCAAACTCAATACAA-3'
ICR-MA-3A30	5'-CACCACCATACTCACAATAATATA-3'
ICRas-MA-3A1	5'-TTAAACCCCAACCTCTACTTTTAT-3'
ICRas-MA-3A3	5'-AACTCCTAATAATTCATTTACATTTC-3'
H19-MA-3A4	5'-CCACTACTAAATAATCATAACTAATCAA-3'
H19-MA-3A6	5'-AAACAATACCAAACCCTATCTAAA-3'
H19as-MA-3A1	5'-ATCACATTAACCACACCTATCATC-3'
H19as-MA-3A2	5'-AATAAAACTACTTCCAAACTAAAC-3'
H19as-MA-3A3	5'-ACTCTATCAACGTATCAATACATAACCC-3'

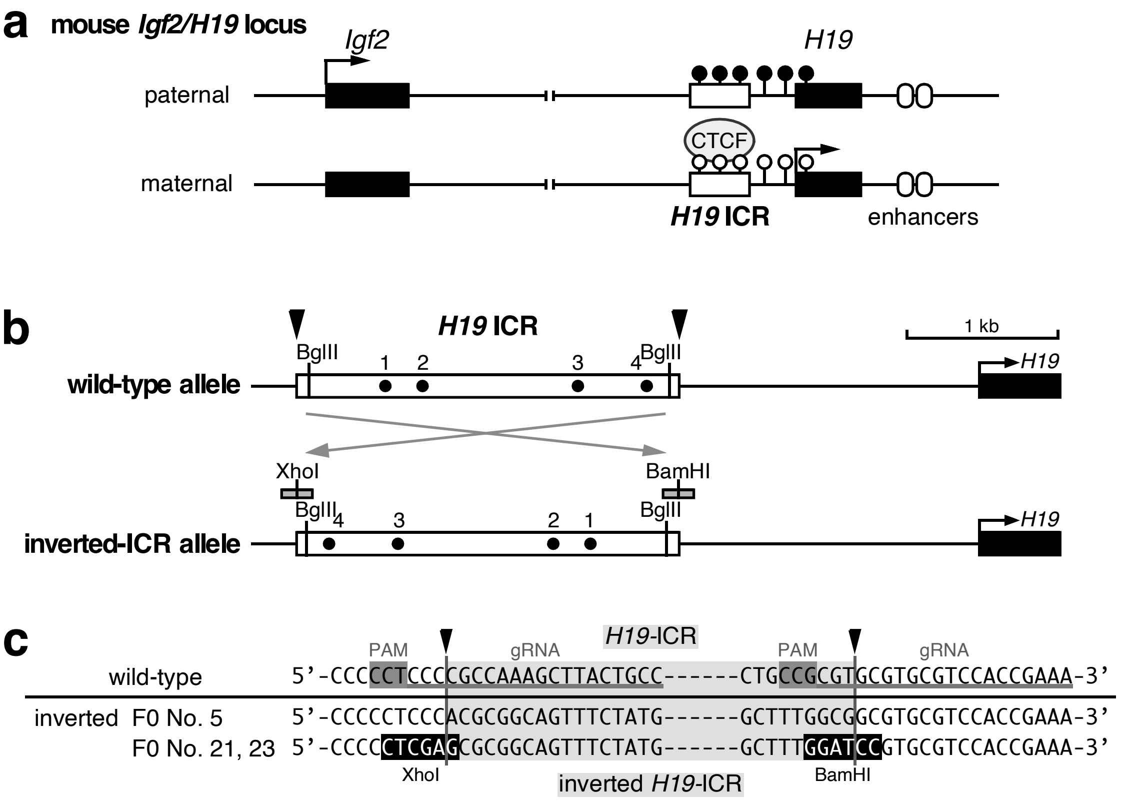
a
mouse Igf2/H19 locus
Igf2
H19
paternal
maternal
CTCF
H19 ICR
enhancers
b
H19 ICR
1 kb
BglII
BglII
1
2
3
4
H19
wild-type allele
XhoI
BamHI
BglII
BglII
4
3
2
1
H19
inverted-ICR allele
c
H19-ICR
PAM
gRNA
PAM
gRNA
wild-type
5’-CCCCCTCCCCGCCAAAGCTTACTGCC------CTGCCGCGTGCGTGCGTCCACCGAAA-3’
inverted F0 No. 5
5’-CCCCCTCCCACGCGGCAGTTTCTATG------GCTTTGGCGGCGTGCGTCCACCGAAA-3’
F0 No. 21, 23
5’-CCCCCTCGAGCGCGGCAGTTTCTATG------GCTTTGGATCCGTGCGTCCACCGAAA-3’
XhoI
inverted H19-ICR
BamHI

Supplementary Figure 1. Generation of the *H19* ICR-inverted mice.

(a) Structure of the mouse endogenous *Igf2/H19* locus. The expression of *Igf2* and *H19* genes depends on the shared 3' enhancer and DNA methylation status of the *H19* ICR, which is located approximately at − 4 to − 2 kb relative to the transcription start site of *H19* gene. Closed and open lollipops indicate methylated and unmethylated CpGs, respectively.

(b) Experimental scheme for generating the inverted-ICR allele. Guide RNAs were designed to digest both side of the *H19* ICR (arrowheads; 2.5 kb apart). In addition to expression vectors for Cas9 protein and guide RNAs, ssODNs (gray rectangles), the sequences of which were corresponding to junction region after inversion, were introduced in mouse zygotes to induce *H19* ICR inversion. Dots (1–4) indicate CTCF-binding sites.

(c) Sequence alignment of wild-type (reference) and mutant alleles confirmed the inverted-ICR alleles. Protospacer-adjacent motif (PAM) and gRNA sequences are shaded and underlined, respectively. Cleavage sites predicted from PAM locations are indicated by arrowheads. When ssODNs are used for recombination, *Xho*I (at 5' junction) or *Bam*HI (at 3' junction) sites are introduced at the junctions.

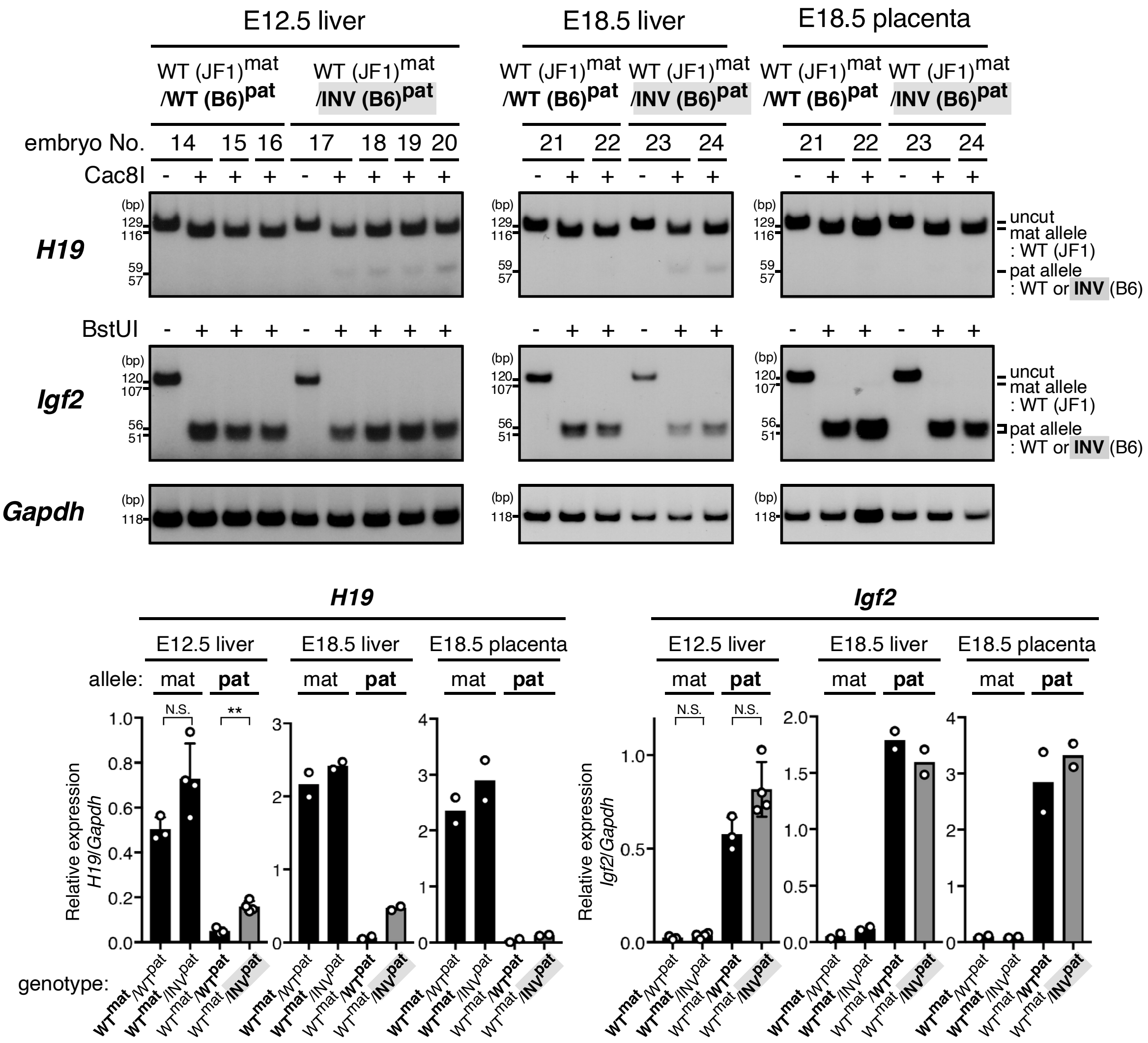
E12.5 liver
E18.5 liver
E18.5 placenta
WT (JF1)mat /WT (B6)pat
WT (JF1)mat /INV (B6)pat
embryo No.
Cac8I
BstUI
H19
Igf2
Gapdh
uncut
mat allele : WT (JF1)
pat allele : WT or INV (B6)
allele: mat pat
Relative expression H19/Gapdh
Relative expression Igf2/Gapdh
N.S.
**
genotype:

Supplementary Figure 2. The *H19* and *Igf2* genes expression analysis in embryos in which the inverted ICR alleles were paternally inherited.

Total RNA was prepared from embryonic tissues from a litter which was distinct from that of Fig. 1, and allele-specific expression analysis was performed as described in the legend to Fig. 1. **p < 0.01.

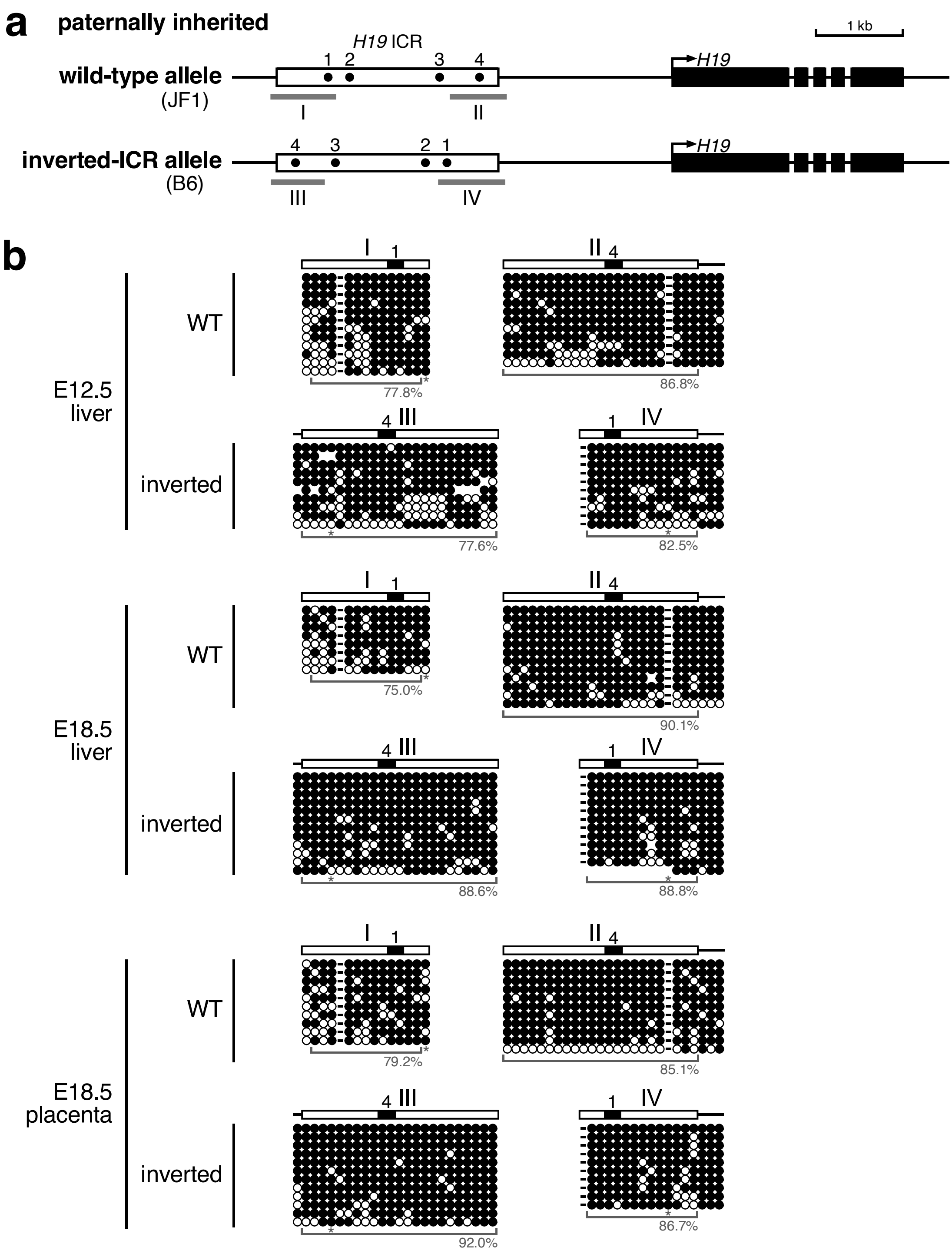
a
paternally inherited
H19 ICR
1 2 3 4
wild-type allele
(JF1)
H19
I
II
1 kb
4 3 2 1
inverted-ICR allele
(B6)
H19
III
IV
b
E12.5
liver
WT
inverted
I 1
II 4
77.8%
86.8%
4 III
1 IV
77.6%
82.5%
E18.5
liver
75.0%
90.1%
88.6%
88.8%
E18.5
placenta
79.2%
85.1%
92.0%
86.7%

Supplementary Figure 3. DNA methylation was acquired properly on the paternally inherited inverted-*H19* ICR in fetal tissues.

(a) Map of wild-type and inverted-ICR alleles. Regions analyzed by bisulfite sequencing in (b) were indicated by gray bars below the map.

(b) DNA methylation status of the wild-type and inverted *H19* ICR in livers (E12.5 and E18.5) and placentas (E18.5). For analyses of paternally inherited wild-type (WT) allele, genomic DNA of tissues from INV(B6)^{mat}/WT(JF1)^{pat} embryos which were identical to ones analyzed in Fig. 3c (E12.5, No. 5-7; E18.5, No. 13-16) were pooled. For analyses of paternally inherited inverted-ICR allele, genomic DNA of tissues from WT(JF1)^{mat}/INV(B6)^{pat} embryos which were identical to ones in Fig. 1c (E12.5, No. 4-6; E18.5, No.12-13) were pooled. Parental origin of the alleles was discriminated by using allele specific (*i.e.* WT or inverted) PCR primer sets and confirmed by SNPs between B6 and JF1. Each horizontal row represents a single DNA template molecule. Methylated and unmethylated CpG motifs are shown as filled and open circles, respectively. Position of CTCF-binding sites is shown by filled boxes. The methylation levels (%) of CpGs inside the *H19* ICR excluding allele-specific sites (*) are shown for each cluster.

Supplementary Figure 4

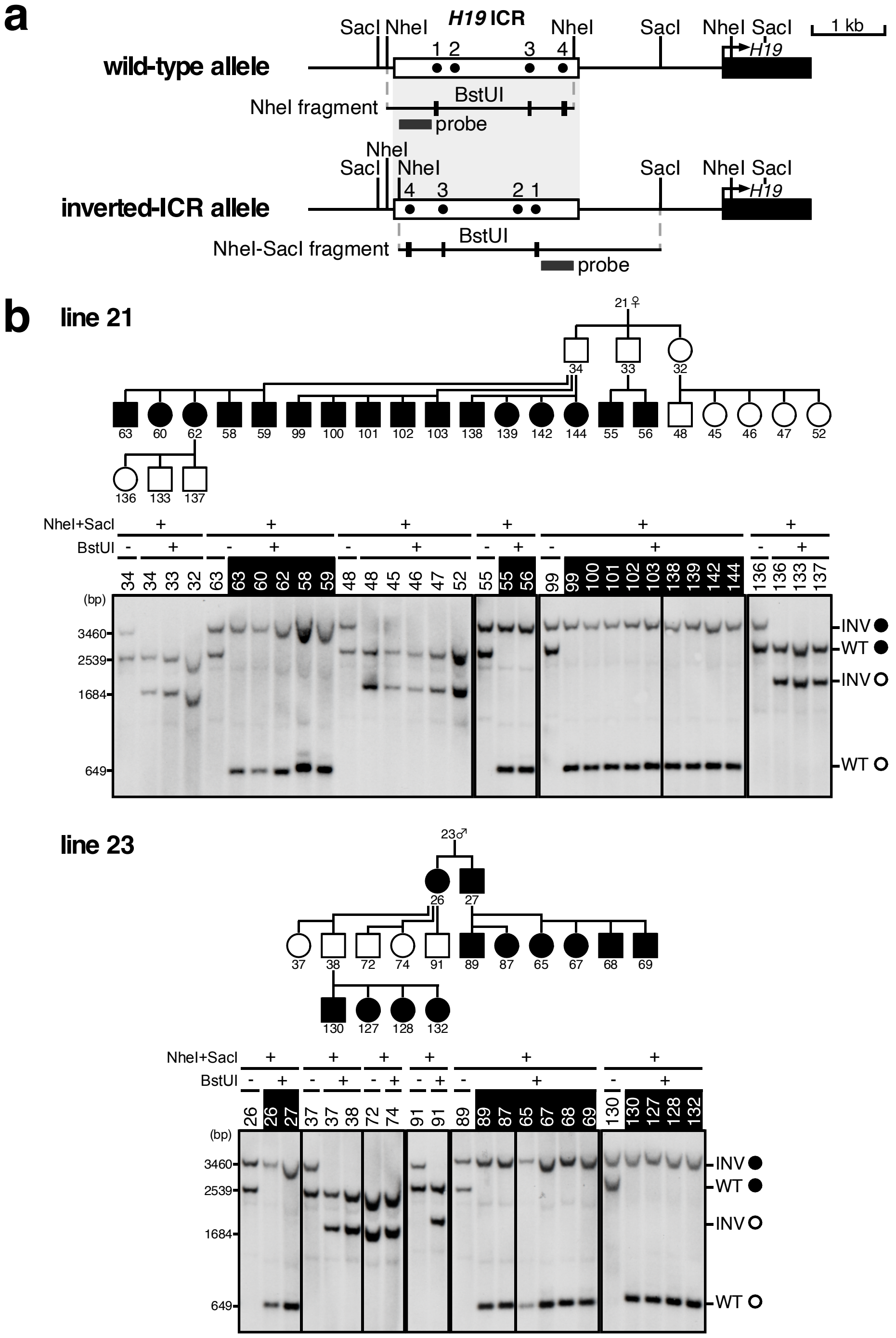

Supplementary Figure 4. Inverted *H19* ICR was differentially methylated in tail somatic cells.

(a) Partial restriction enzyme maps of the *H19* locus carrying either the wild-type (top) or inverted (bottom) *H19* ICRs. Methylation-sensitive *Bst*UI sites in *Nhe*I (wild-type) or *Nhe*I-*Sac*I (inverted-ICR) fragments are displayed as vertical lines beneath each map.

(b) DNA methylation status of the *H19* gene locus in tail somatic cells of inverted-ICR heterozygous mice. Tail genomic DNA was digested with *Nhe*I+*Sac*I alone or with *Bst*UI and the Southern blots were hybridized with the probe shown in the maps (a). In the pedigree, male and female individuals carrying the inverted-ICR allele heterozygously are represented as rectangles and circles, respectively. Filled or open symbols indicate hyper- or hypo-methylated status of the inverted-ICR fragment in each mouse, which was independently determined by visual examination of the Southern blot results by three individuals. Filled and open circles on the right of the Southern blot results indicate the positions of parental or methylated and complete digests, respectively.

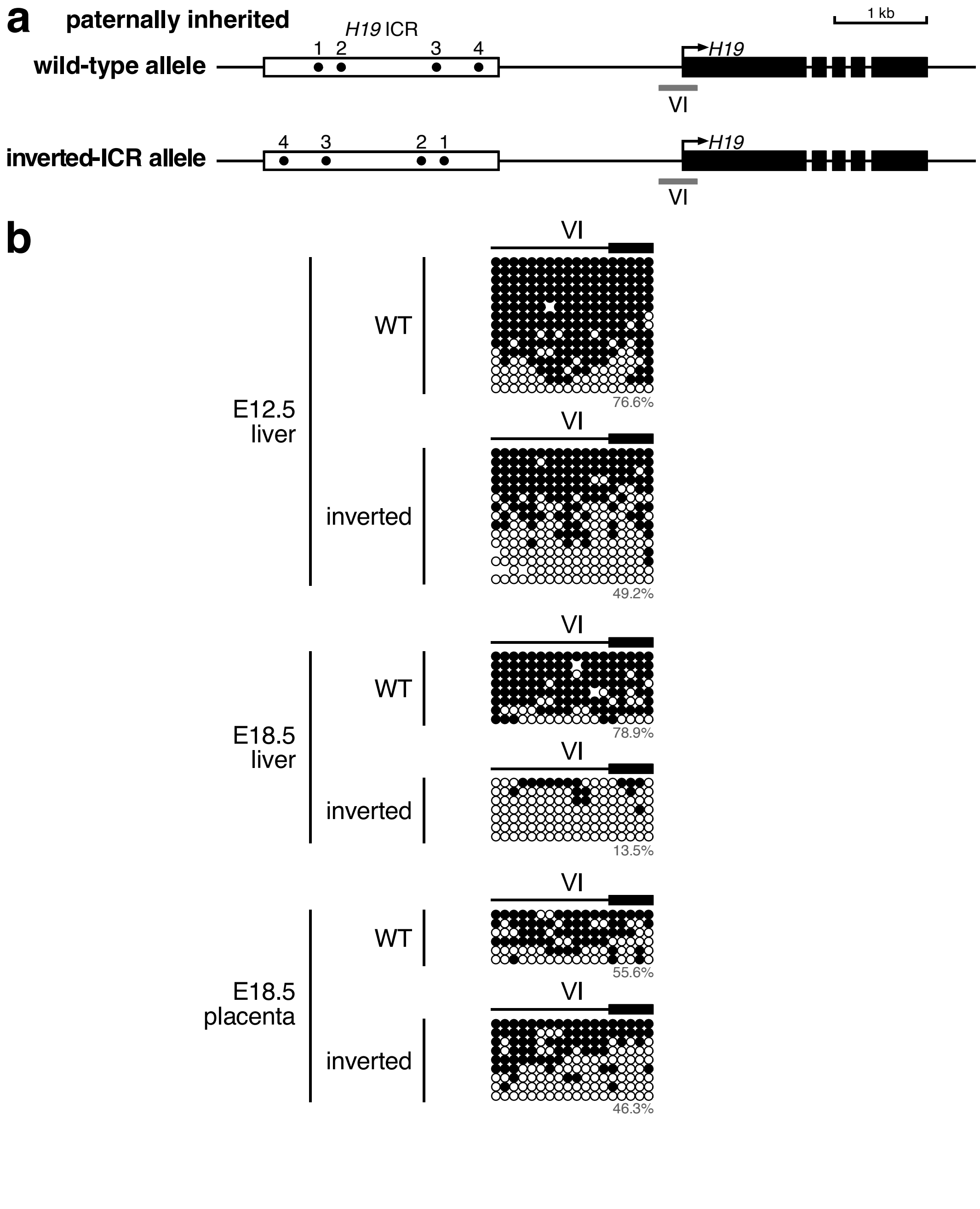
a
paternally inherited
H19 ICR
1 kb
wild-type allele
1 2 3 4
H19
VI
inverted-ICR allele
4 3 2 1
H19
VI
b
E12.5 liver
WT
VI
76.6%
inverted
VI
49.2%
E18.5 liver
WT
VI
78.9%
inverted
VI
13.5%
E18.5 placenta
WT
VI
55.6%
inverted
VI
46.3%

Supplementary Figure 5. DNA methylation status of the paternally inherited *H19* promoter.

(a) Map of wild-type and inverted-ICR alleles. Regions analyzed by bisulfite sequencing in (b) were indicated by gray bars below the map.

(b) DNA methylation status of the wild-type and inverted *H19* ICR alleles in livers (E12.5 and E18.5) and placentas (E18.5). For analyses of paternally inherited wild-type (WT) allele, genomic DNA of tissues from $WT(JF1)^{mat}$/$\underline{WT(B6)^{pat}}$ embryos which were identical to ones analyzed in Supplementary Fig. 2 (E12.5, No. 14-16; E18.5, No. 21-22) were pooled. For analyses of paternally inherited inverted-ICR allele, genomic DNA of tissues from $WT(JF1)^{mat}$/$\underline{INV(B6)^{pat}}$ embryos which were identical to ones in Supplementary Fig. 2 (E12.5, No. 17-20; E18.5, No. 23-24) were pooled. Parental origin of the alleles was discriminated by using WT or inverted-allele specific PCR primer sets and by SNPs between B6 and JF1. The methylation levels (%) are shown for each cluster and were statistically compared (WT vs inverted, $p = 0.0347$ [E12.5 liver], $p = 0.0006$ [E18.5 liver], $p = 0.6268$ [E18.5 placenta]).

Supplementary Figure 6

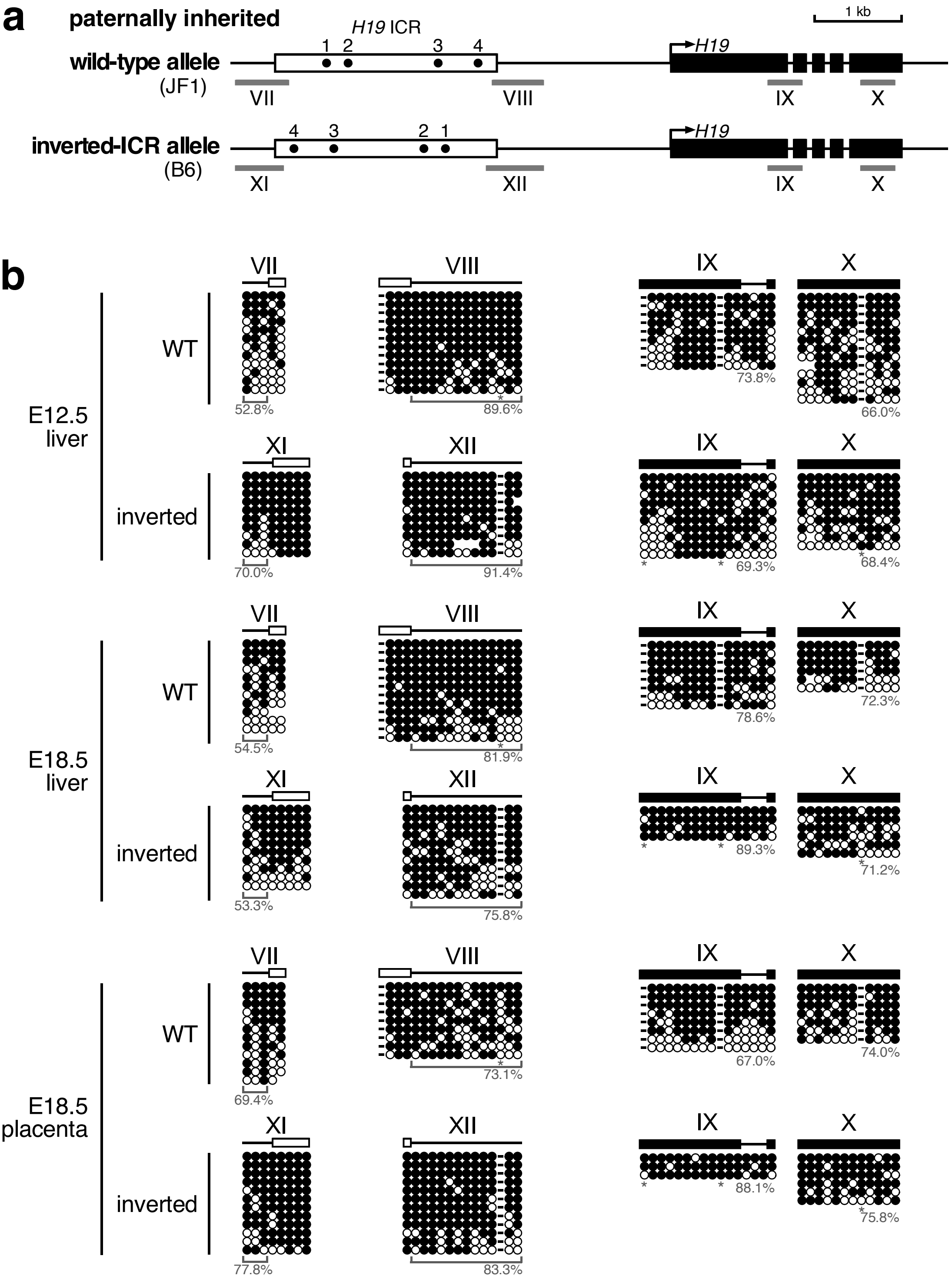

Supplementary Figure 6. Methylation status of the paternally inherited *H19* locus in fetal tissues.

(a) Map of wild-type and inverted-ICR alleles. Regions indicated by gray bars below the map were analyzed by bisulfite sequencing in (b).

(b) DNA methylation status of the paternally inherited wild-type and inverted *H19* ICR alleles in livers (E12.5 and E18.5) and placentas (E18.5) was determined as described in the legend to Supplementary Fig. 3b. There was no significant difference in methylation levels of analyzed regions between wild-type and inverted alleles.

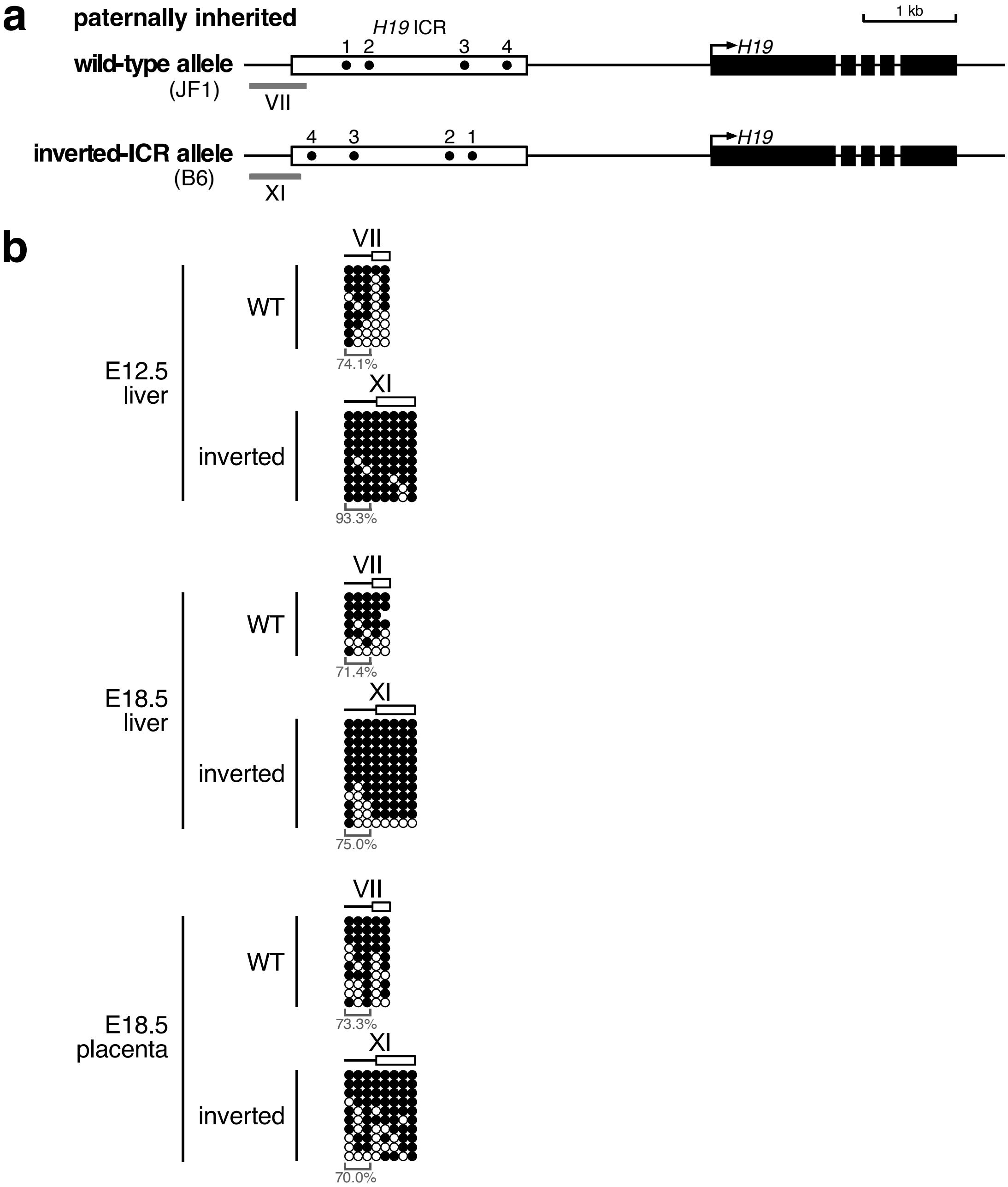
a
paternally inherited
wild-type allele
(JF1)
H19 ICR
1 2
3
4
H19
1 kb
VII
inverted-ICR allele
(B6)
4
3
2 1
XI
b
E12.5
liver
WT
inverted
74.1%
93.3%
E18.5
liver
71.4%
75.0%
E18.5
placenta
73.3%
70.0%

Supplementary Figure 7. Methylation status of the ICR upstream region on the paternally inherited allele.

(a) Map of wild-type and inverted-ICR alleles. Regions indicated by gray bars below the map were analyzed by bisulfite sequencing in (b).

(b) DNA methylation status of the paternally inherited wild-type and inverted *H19* ICR alleles was determined as described in the legend to Supplementary Fig. 5b. There was no significant difference in methylation levels of analyzed regions between wild-type and inverted alleles.

Supplementary Figure 8

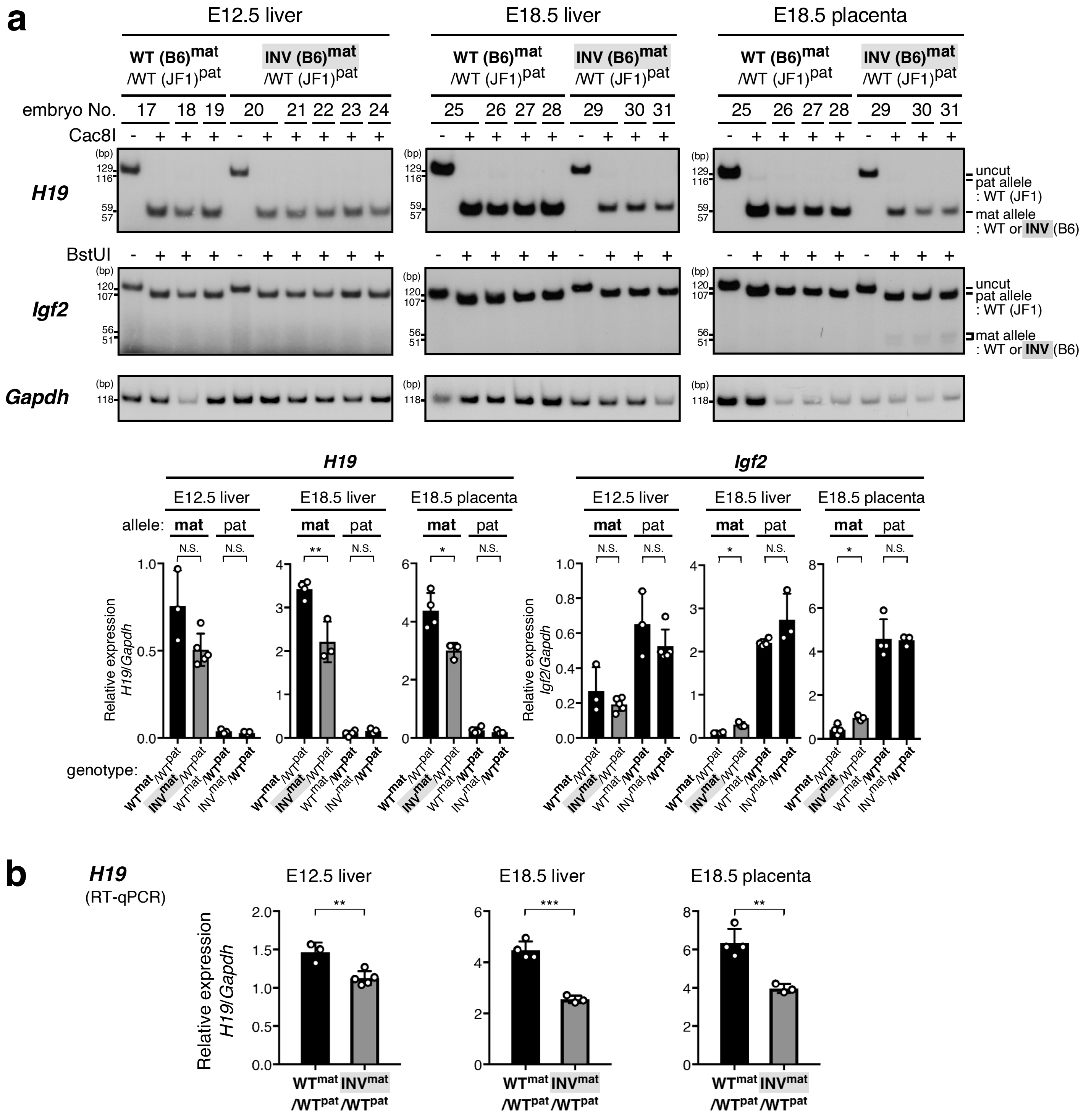

Supplementary Figure 8. The *H19* and *Igf2* genes expression analysis in embryos in which the inverted ICR alleles were maternally inherited.

Total RNA was prepared from embryonic tissues from a litter which was distinct from that of Fig. 3. Allele-specific expression analysis (in (a)) and quantification of the *H19* gene expression levels (in (b)) were performed as described in the legend to Fig. 3. The means±SD was displayed on the graph (*$p < 0.05$, **$p < 0.01$, ***$p < 0.001$).

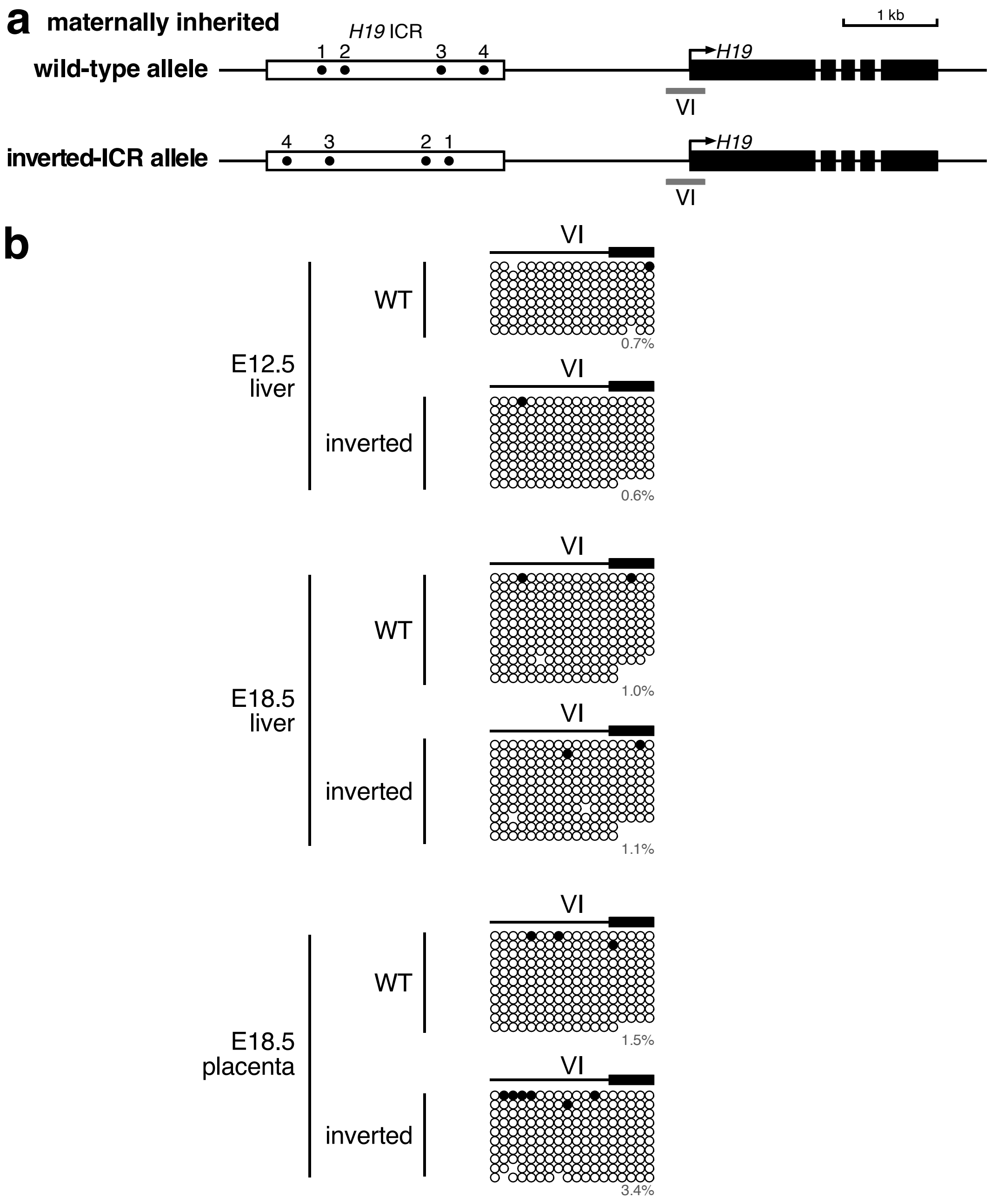
a
maternally inherited
H19 ICR
1 kb
1 2 3 4
H19
wild-type allele
VI
4 3 2 1
H19
inverted-ICR allele
VI
b
E12.5 liver
WT
inverted
VI
0.7%
0.6%
E18.5 liver
WT
inverted
1.0%
1.1%
E18.5 placenta
WT
inverted
1.5%
3.4%

Supplementary Figure 9. Methylation status of the maternally inherited *H19* promoter in fetal tissues.

(a) Map of wild-type and inverted-ICR alleles. Regions indicated by gray bars below the map were analyzed by bisulfite sequencing in (b).

(b) For analyses of maternally inherited wild-type (WT) allele, genomic DNA of tissues from $\underline{\text{WT(B6)}^{\text{mat}}}$/WT(JF1)^{pat} embryos which were identical to ones analyzed in Supplementary Fig. 8 (E12.5, No. 17-19; E18.5, No. 25-28) were pooled. For analyses of maternally inherited inverted allele, genomic DNA of tissues from $\underline{\text{INV(B6)}^{\text{mat}}}$/WT(JF1)^{pat} embryos which were identical to ones in Supplementary Fig. 8 (E12.5, No. 20-24; E18.5, No. 29-31) were pooled. Parental origin of the alleles was determined by SNPs between B6 and JF1. Each horizontal row represents a single DNA template molecule. Methylated and unmethylated CpG motifs are shown as filled and open circles, respectively. The methylation levels (%) are shown for each cluster. There was no significant difference in methylation levels between wild-type and inverted alleles.

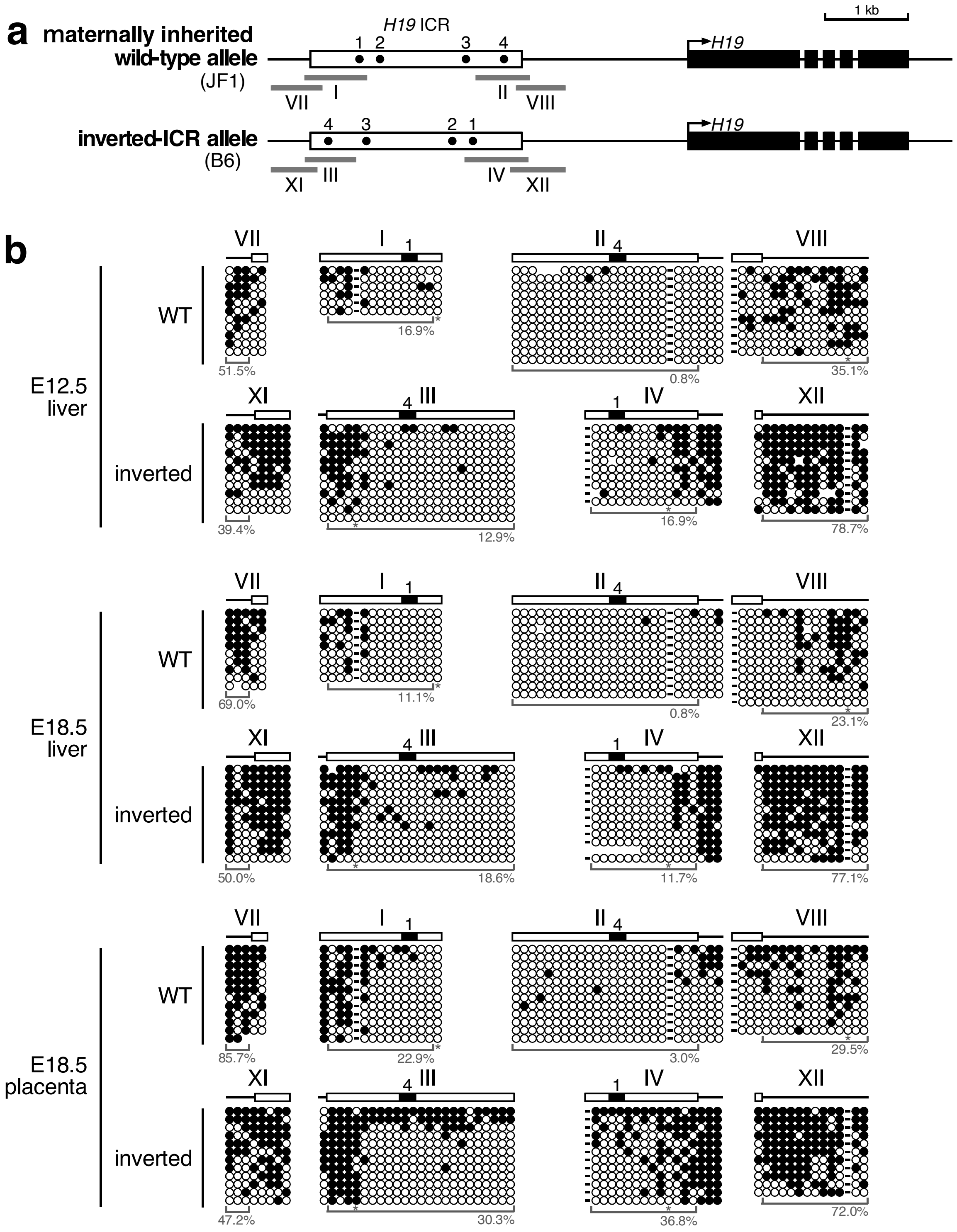

a
maternally inherited wild-type allele (JF1)
H19 ICR
1 2 3 4
H19
1 kb
VII I II VIII
inverted-ICR allele (B6)
4 3 2 1
H19
XI III IV XII
b
E12.5 liver
WT
VII 51.5%
I 1 16.9%
II 4 0.8%
VIII 35.1%
inverted
XI 39.4%
4 III 12.9%
1 IV 16.9%
XII 78.7%
E18.5 liver
WT
VII 69.0%
I 1 11.1%
II 4 0.8%
VIII 23.1%
inverted
XI 50.0%
4 III 18.6%
1 IV 11.7%
XII 77.1%
E18.5 placenta
WT
VII 85.7%
I 1 22.9%
II 4 3.0%
VIII 29.5%
inverted
XI 47.2%
4 III 30.3%
1 IV 36.8%
XII 72.0%

Supplementary Figure 10. Methylation status of the maternally inherited *H19* ICR and its surrounding sequences in fetal tissues.

(a) Map of wild-type and inverted-ICR alleles. Regions indicated by gray bars below the map were analyzed by bisulfite sequencing in (b).

(b) DNA methylation status of the maternally inherited wild-type and inverted *H19* ICR alleles in livers (E12.5 and E18.5) and placentas (E18.5) was determined as described in the legend to Fig. 4b. Position of CTCF-binding sites is shown by filled boxes. The methylation levels (%) of CpGs excluding allele-specific sites (*) are shown for each cluster (VIII (WT) vs XII (inverted), $p = 0.0002$ [E12.5 liver], $p = 0.0001$ [E18.5 liver], $p = 0.0007$ [E18.5 placenta]).

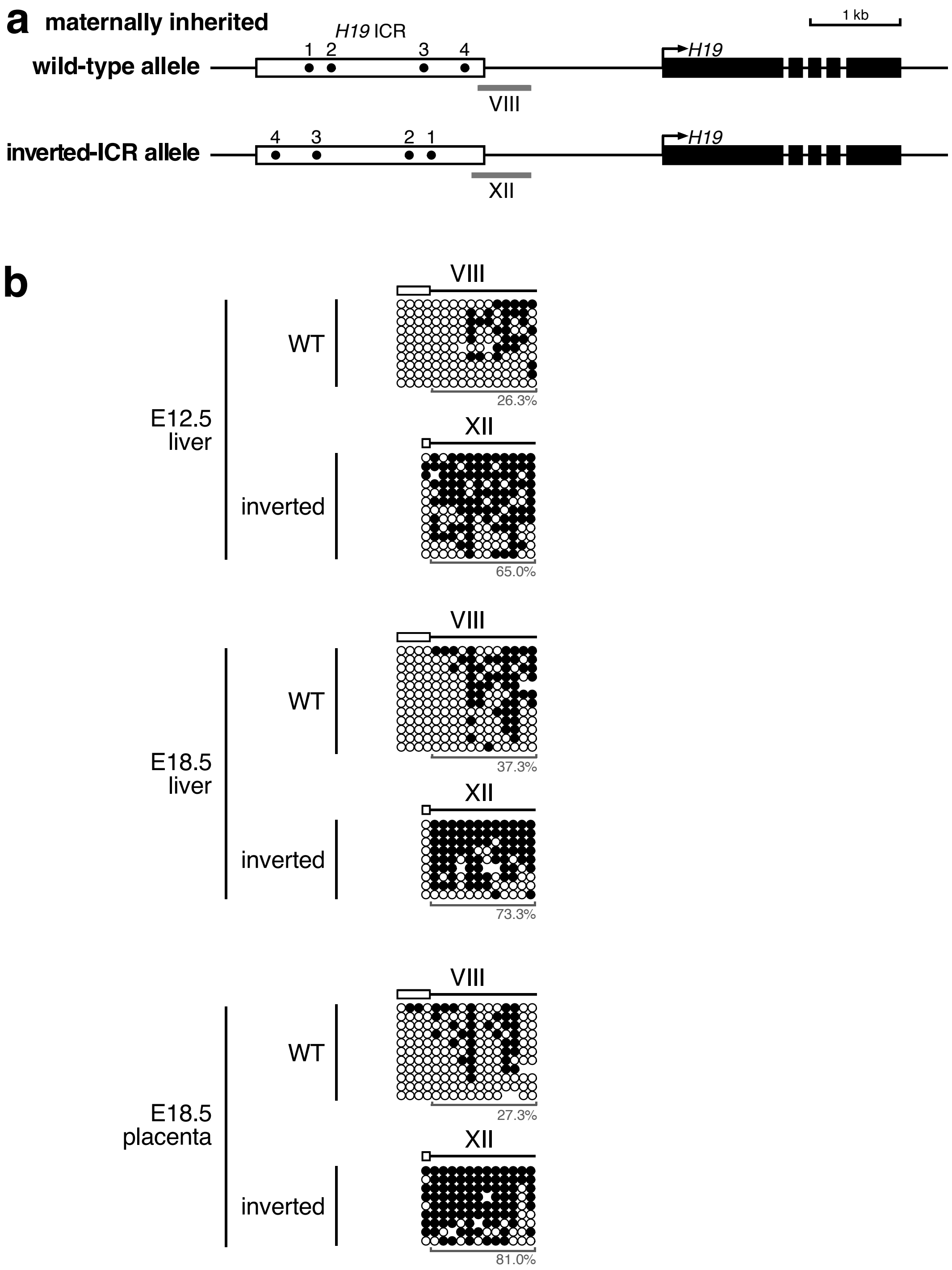
a
maternally inherited
H19 ICR
1 kb
wild-type allele
1 2 3 4
H19
VIII
inverted-ICR allele
4 3 2 1
H19
XII
b
E12.5
liver
WT
VIII
26.3%
inverted
XII
65.0%
E18.5
liver
WT
VIII
37.3%
inverted
XII
73.3%
E18.5
placenta
WT
VIII
27.3%
inverted
XII
81.0%

Supplementary Figure 11. Methylation status of the ICR downstream region on the maternally inherited allele.

(a) Map of wild-type and inverted-ICR alleles. Regions indicated by gray bars below the map were analyzed by bisulfite sequencing in (b).

(b) DNA methylation status of the maternally inherited wild-type and inverted *H19* ICR alleles was determined as described in the legend to Supplementary Fig. 9b. The methylation levels (%) are shown for each cluster (VIII (WT) vs XII (inverted), p = 0.0008 [E12.5 liver], p = 0.0053 [E18.5 liver], p = 0.0000 [E18.5 placenta]).

Supplementary Figure 12

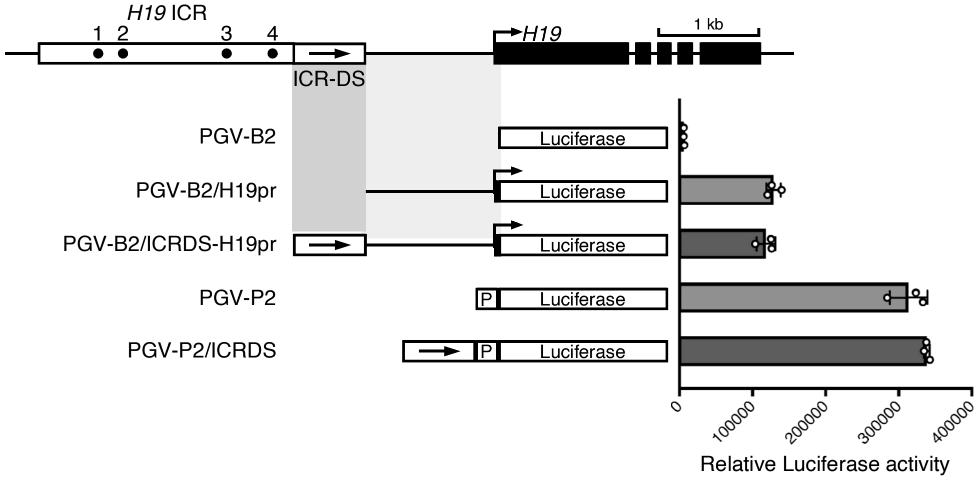

Supplementary Figure 12. Verification of transcriptional regulatory activity in the *H19* ICR downstream region.

ICR-DS sequence was linked to the luciferase reporter gene under the control of the *H19* gene (PGV-B2/ICRDS-H19pr) or SV40 (PGV-P2/ICRDS) promoter. These reporter constructs, as well as the control plasmid CMV-β-Gal, were cotransfected into MEF cells. Each value represents the mean±SD for three technical replicates.

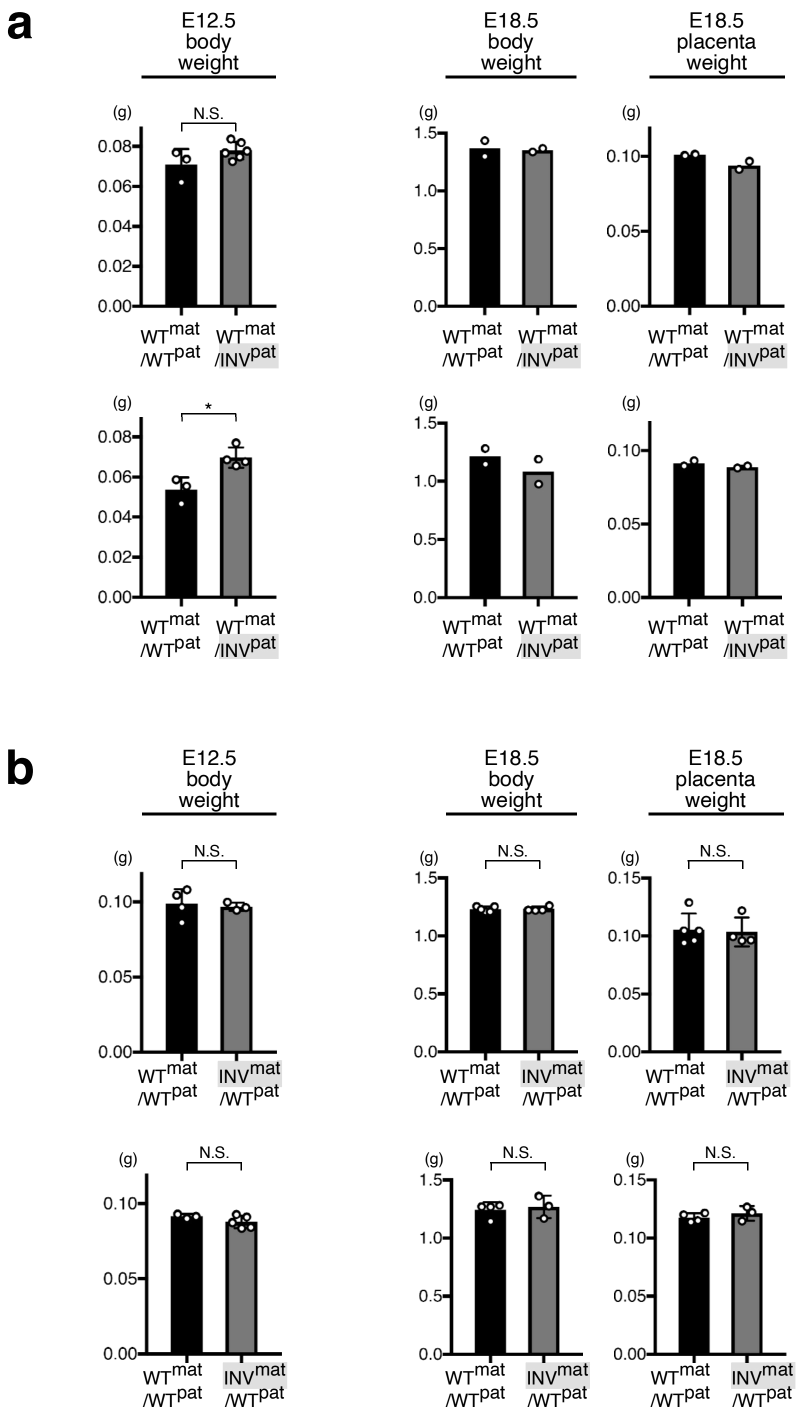
a
E12.5 body weight
E18.5 body weight
E18.5 placenta weight
(g)
N.S.
*
0.08
0.06
0.04
0.02
0.00
1.5
1.0
0.5
0.0
0.10
0.05
0.00
WTmat /WTpat
WTmat /INVpat
b
E12.5 body weight
E18.5 body weight
E18.5 placenta weight
(g)
N.S.
0.15
0.10
0.05
0.00
1.5
1.0
0.5
0.0
WTmat /WTpat
INVmat /WTpat

Supplementary Figure 13. Body and placental weight of embryos.

Weight of embryos (E12.5 and E18.5) and placentas (E18.5) which are analyzed in Fig. 1 (upper in (a)), Supplementary Fig. 2 (lower in (a)), Fig. 3 (upper in (b)), and Supplementary Fig. 8 (lower in (b)) are shown (the means±SD, $*p < 0.05$).

Supplementary Figure 14

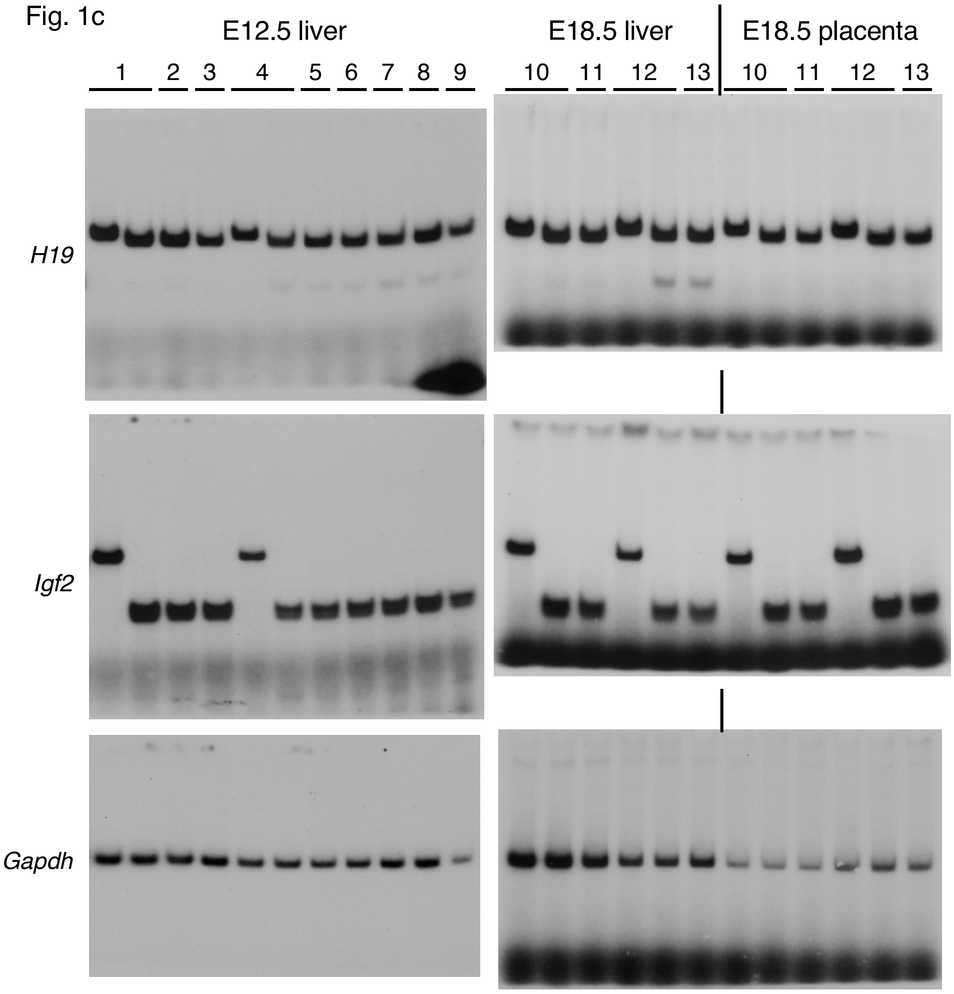

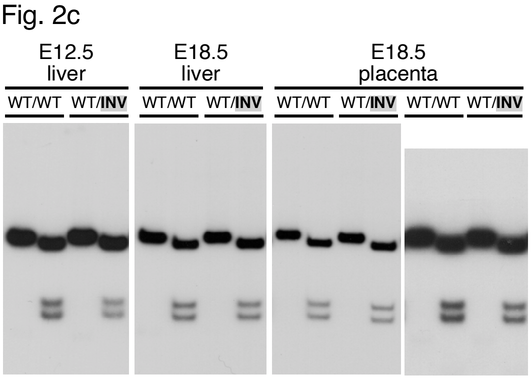

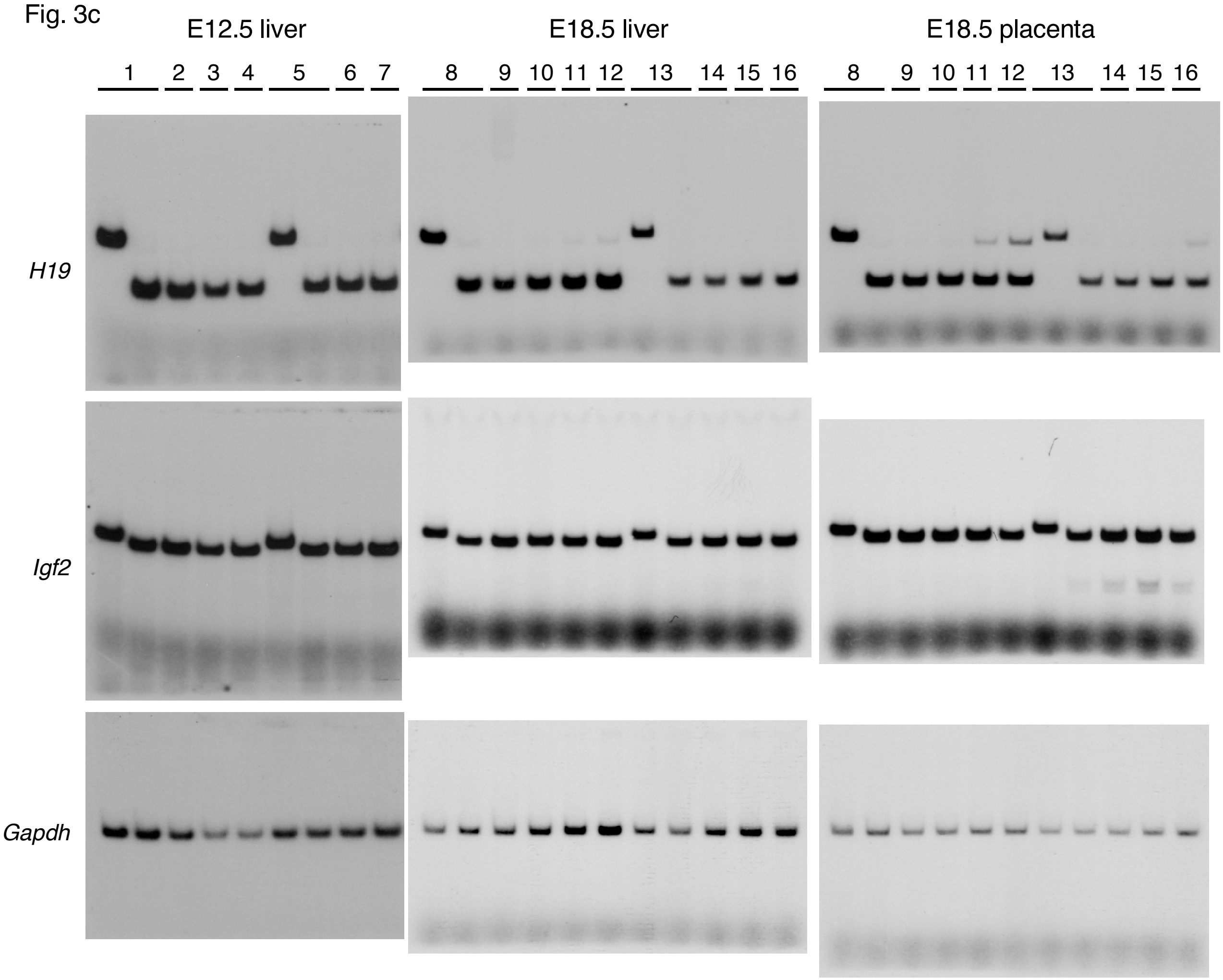

Supplementary Figure 14. Uncropped and unedited images.

Supplementary Fig. 2

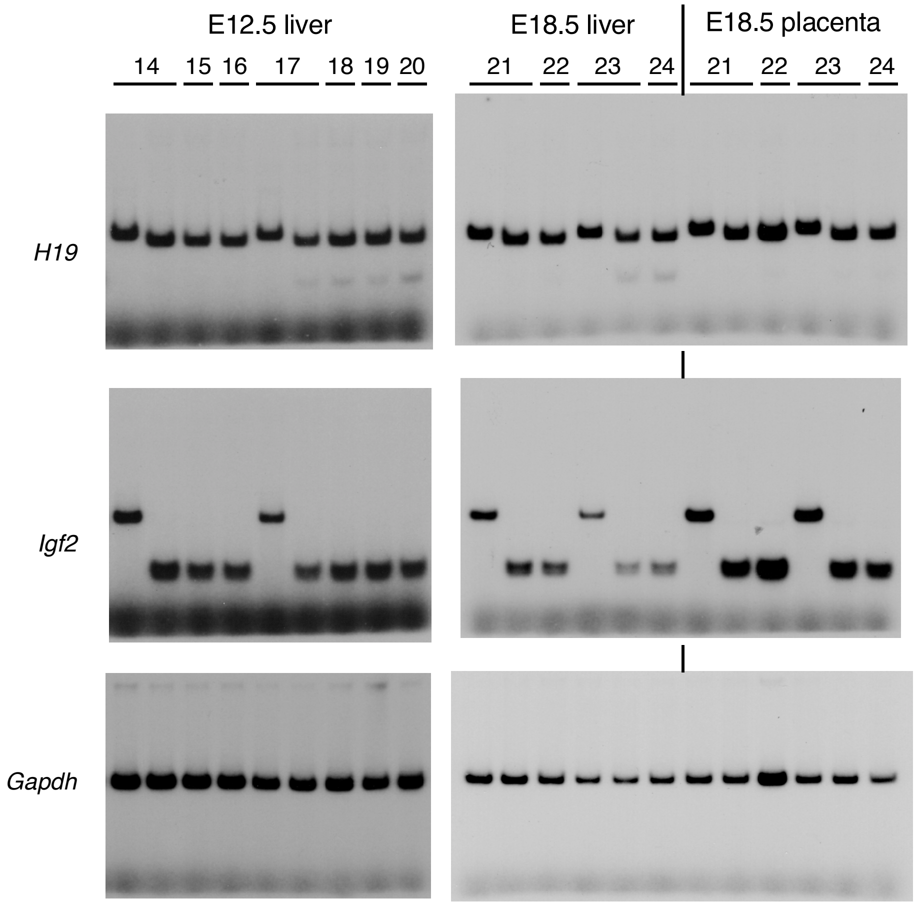

Supplementary Fig. 4

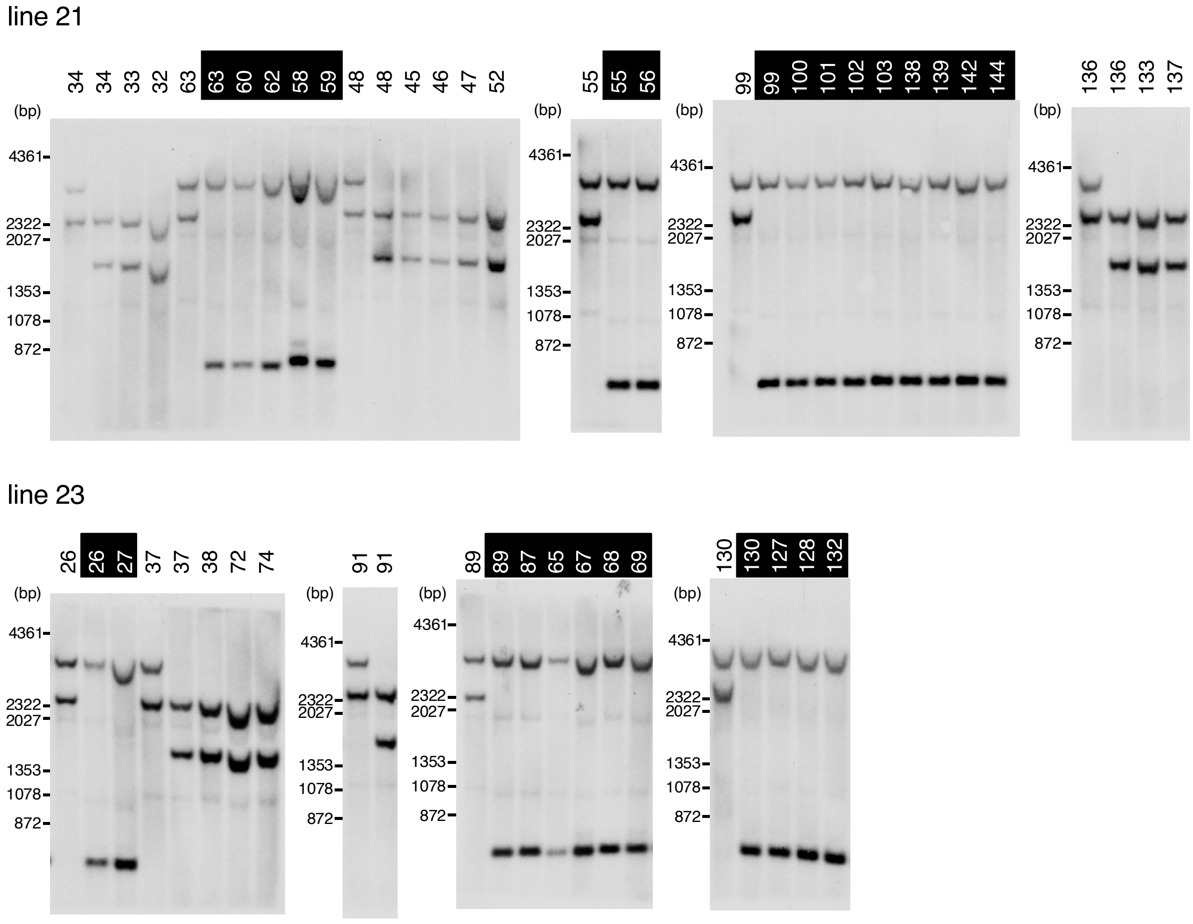

Supplementary Figure 15. Uncropped and unedited images.

Supplementary Fig. 8

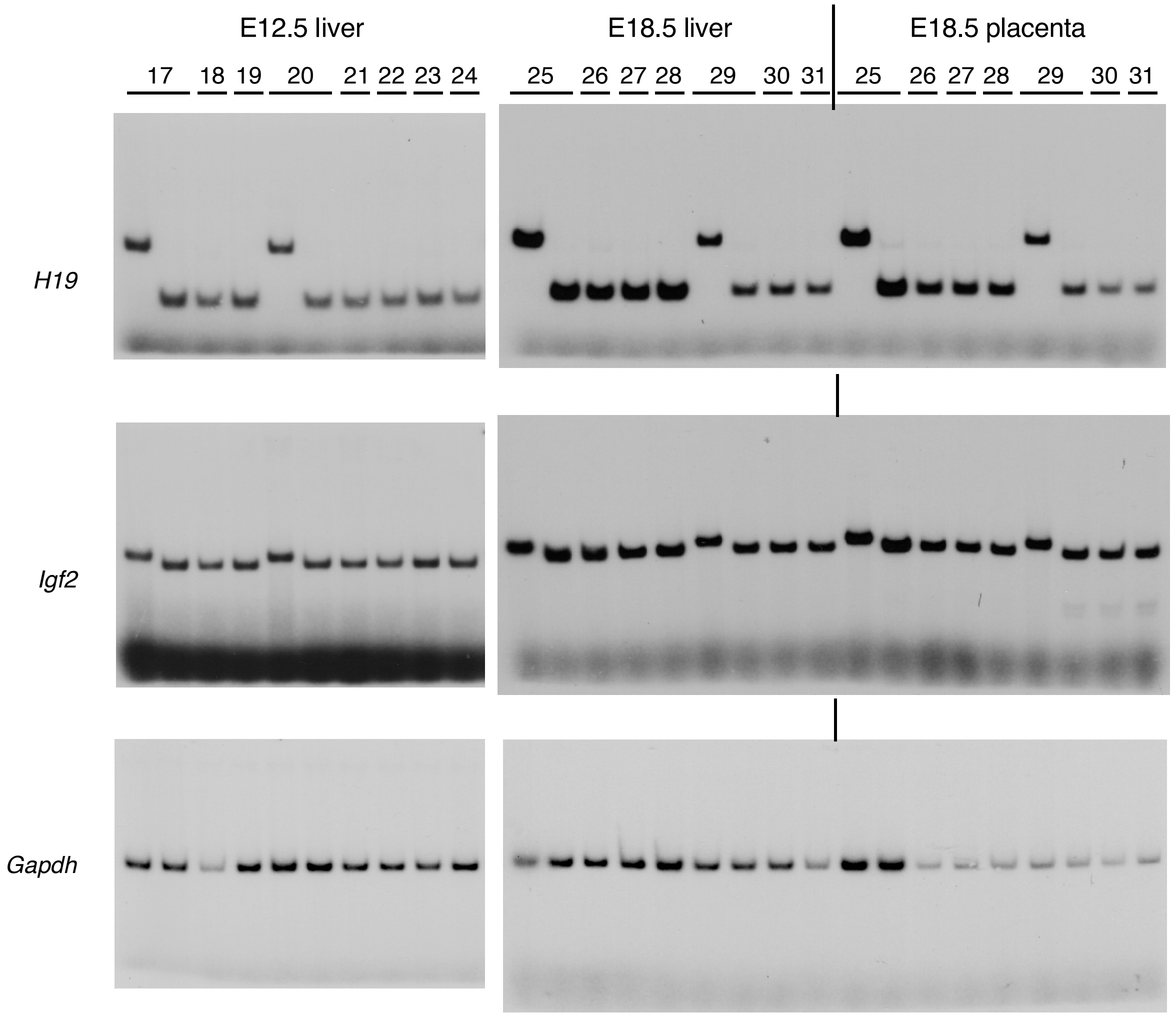

Supplementary Figure 16. Uncropped and unedited images.
