# Supplementary material for: Orientation of mouse H19 ICR affects imprinted H19 gene expression through promoter methylation-dependent and -independent mechanisms
Source: Commun Biol. 2021 Dec 17;4:1410. doi: 10.1038/s42003-021-02939-9 (PMC8683476; doi:10.1038/s42003-021-02939-9)
Supplement: Supplementary file 2 — Description of Additional Supplementary Files [file 42003_2021_2939_MOESM2_ESM.pdf]

Description of Additional Supplementary Files

File name: Supplementary Data 1.
Description: Source data for graphs.
